# Highly efficient prime editing by introducing same-sense mutations in pegRNA or stabilizing its structure

**DOI:** 10.1038/s41467-022-29339-9

**Published:** 2022-03-29

**Authors:** Xiaosa Li, Lina Zhou, Bao-Qing Gao, Guangye Li, Xiao Wang, Ying Wang, Jia Wei, Wenyan Han, Zixian Wang, Jifang Li, Runze Gao, Junjie Zhu, Wenchao Xu, Jing Wu, Bei Yang, Xiaodong Sun, Li Yang, Jia Chen

**Affiliations:** 1 Department of Ophthalmology, Shanghai General Hospital, Shanghai Jiao Tong University School of Medicine, 200080 Shanghai, China; 2 Shanghai Key Laboratory of Fundus Diseases, 200080 Shanghai, China; 3 Gene Editing Center, School of Life Science and Technology, ShanghaiTech University, 201210 Shanghai, China; 4 Center for Excellence in Molecular Cell Science, Shanghai Institute of Biochemistry and Cell Biology, Chinese Academy of Sciences, 200031 Shanghai, China; 5 University of Chinese Academy of Sciences, 100049 Beijing, China; 6 Shanghai Clinical Research and Trial Center, 201210 Shanghai, China; 7 CAS Key Laboratory of Computational Biology, Shanghai Institute of Nutrition and Health, University of Chinese Academy of Sciences, Chinese Academy of Sciences, 200031 Shanghai, China; 8 Shanghai Institute for Advanced Immunochemical Studies, ShanghaiTech University, Shanghai, 201210 China; 9 Center for Molecular Medicine, Children’s Hospital, Fudan University and Shanghai Key Laboratory of Medical Epigenetics, International Laboratory of Medical Epigenetics and Metabolism, Ministry of Science and Technology, Institutes of Biomedical Sciences, Fudan University, Shanghai, 200032 China

**Keywords:** Genetic engineering, CRISPR-Cas9 genome editing

## Abstract

Prime editor (PE), which is developed by combining Cas9 nickase and an engineered reverse transcriptase, can mediate all twelve types of base substitutions and small insertions or deletions in living cells but its efficiency remains low. Here, we develop spegRNA by introducing same-sense mutations at proper positions in the reverse-transcription template of pegRNA to increase PE’s base-editing efficiency up-to 4,976-fold (on-average 353-fold). We also develop apegRNA by altering the pegRNA secondary structure to increase PE’s indel-editing efficiency up-to 10.6-fold (on-average 2.77-fold). The spegRNA and apegRNA can be combined to further enhance editing efficiency. When spegRNA and apegRNA are used in PE3 and PE5 systems, the efficiencies of sPE3, aPE3, sPE5 and aPE5 systems are all enhanced significantly. The strategies developed in this study realize highly efficient prime editing at certain previously uneditable sites.

## Abstract (web summary)

Prime editors can mediate all twelve types of base substitutions and small insertions or deletions in living cells but its efficiency remains low. Here the authors introduce same-sense mutations into pegRNAs to increase base-editing efficiency and the pegRNA secondary structure was altered to increase indel-editing efficiency.

## Introduction

PE combines a Cas9 nickase and a reverse transcriptase to integrate the edits encoded in the reverse transcription-template (RTT) of prime editing guide RNA (pegRNA) into targeted genomic DNA^1^, which achieves versatile editing, i.e., all 12 types of base substitutions and small indels in human cells, plants, and fishes^1–3^, with high editing specificity^4–6^. By using a nicking single-guide RNA (sgRNA) in addition to pegRNA, PE3 can trigger endogenous mismatch repair (MMR) to help install its product at on-target sites^1,7^. However, the editing efficiency of PE3 remains generally low^1,8^, which hinders its broad applications.

In this work, we developed a spegRNA strategy by introducing same-sense mutations (SSMs) at proper positions in the RTT of pegRNA to increase PE’s base-editing efficiency or an apegRNA strategy by altering the pegRNA secondary structure to increase PE’s indel-editing efficiency. The spegRNA and apegRNA strategies were successfully applied in PE3^1^ and PE5^9^ systems to induce highly efficient editing across multiple target sites in three types of human cells, and these two strategies can also be combined to further improve PE’s efficiency.

## Results

### Extra point mutation in RTT improves base editing efficiency

**Fig. 1:**
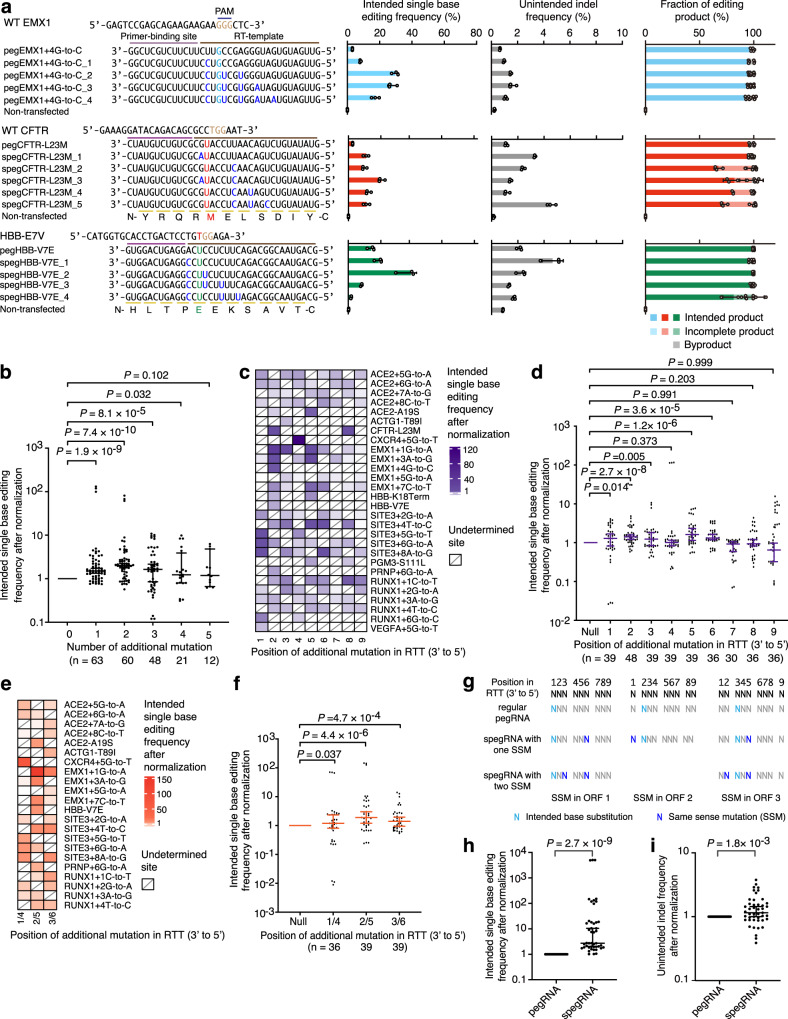
pegRNA containing additional base substitutions induced higher efficiencies of intended single-base editing. **a** Sequences of PBS and RTT of pegRNAs. Intended single-base edits are in cyan, red (pathogenic mutations), or green (corrected base), additional base substitutions are in blue and protospacer adjacent motifs (PAMs) are in brown. The intended single-base editing frequency, unintended indel frequency and fraction of editing product were induced under PE3 setting. Means ± s.d. are from three independent experiments. **b** Statistical analysis of normalized single-base editing frequencies, setting the frequencies induced by regular pegRNAs (without additional base substitution) as 1. *n* = intended single-base editing from three independent experiments in **a** and Supplementary Figs. 1b and 2b, f. **c** Heatmaps show the normalized single-base editing efficiencies induced by the pegRNAs with one additional base substitution, setting the ones induced by regular pegRNAs as 1. **d** Statistical analysis of normalized single-base editing frequencies, setting the frequencies induced by regular pegRNAs as 1. *n* = intended single-base editing from three independent experiments in **c**. **e** Heatmaps show the normalized single-base editing efficiencies induced by the pegRNAs with two additional base substitutions, setting the ones induced by regular pegRNAs as 1. **f** Statistical analysis of normalized single-base editing frequencies, setting the frequencies induced by regular pegRNAs as 1. *n* = intended single-base editing from three independent experiments in **e**. **g** The strategy to design efficient spegRNAs, depending on the relative positions between the ORF of edited gene and the 3’-end of RTT. **h**, **i** Statistical analysis of normalized single-base editing frequencies (**h**) and unintended indel frequencies (**i**) induced by the pegRNAs designed according to the rule shown in **g**, setting the frequencies induced by regular pegRNAs as 1. *n* = 45 intended single-base editing (**h**) and unintended indel editing (**i**) from three independent experiments in Supplementary Fig. 10. The data in **c** and **d** are from **a** and Supplementary Figs. 1b, 2b, e, and 8a. The data in **e** and **f** are from **a** and Supplementary Figs. 1b, 2b, e, and 8b. **b**, **d**, **f**, **h**, **i** *P* value, Wilcoxon one-tailed signed-rank test. The median and interquartile range (IQR) are shown. Source data are provided as a Source Data file.

As an sgRNA is used in the PE3 system to trigger the MMR pathway to install intended edits into targeted genomic loci^1,7^, the editing efficiency of PE3 is associated with endogenous MMR efficiency. Given that the MMR efficiency of correcting single base-base mismatch is usually lower than that of correcting indels^10–12^, we speculate that introducing additional base substitutions into the RTT of pegRNA may enhance the intended base editing efficiency by PE3. We first compared the editing efficiencies induced by regular pegRNAs that contain only an intended single-base substitution with those induced by pegRNAs that contain both the intended single-base substitution and additional base substitutions (Fig. 1a and Supplementary Fig. 1a). As expected, some optimal pegRNAs that contain additional base substitutions (e.g., pegEMX1 + 4G-to-C_2, pegCXCR4 + 5G-to-T_1, pegSITE3 + 5G-to-T_1, pegPNRP+ 6G-to-T_2, pegRUNX1 + 6G-to-C_2 and pegVEGFA+5G-to-T_1 in Fig. 1a and Supplementary Fig. 1a) mediated higher editing efficiencies than their corresponding regular pegRNAs (Fig. 1a and Supplementary Fig. 1b). We also applied additional base substitution-containing pegRNAs to induce single-base substitutions to generate three pathogenic mutations (Fig. 1a and Supplementary Fig. 2a) or correct three preinstalled mutations (Fig. 1a and Supplementary Fig. 2e), which are associated with human diseases. To avoid potential amino acid changes, we introduced SSMs, instead of random mutations, into the RTT of pegRNAs (Fig. 1a and Supplementary Fig. 2a, e). Compared to the relatively low efficiencies induced by regular pegRNAs, some spegRNAs clearly increased the editing efficiencies when generating or correcting pathogenic mutations by PE3 (Fig. 1a and Supplementary Fig. 2b, f).

To further evaluate spegRNA-mediated editing, we also determined the unintended indels, incomplete products (i.e., the products with only SSMs but no intended base editing, Supplementary Fig. 3a–c) and the byproducts (i.e., the products with pegRNA scaffold incorporation, Supplementary Fig. 4a–c) at on-target sites and pegRNA-dependent off-target (OT) editing at predicted sites (Supplementary Fig. 5)^13,14^. The frequencies of unintended indels (Fig. 1a and Supplementary Figs. 1c and 2c, g) and the fractions of incomplete products and byproducts (Fig. 1a and Supplementary Figs. 1d and 2d, h) were not significantly affected when comparing the use of spegRNAs to the use of regular pegRNAs. In addition, no observable OT editing was induced by spegRNA at predicted pegRNA-dependent OT sites (Supplementary Fig. 5a–c). As SSMs may affect gene function by altering mRNA splicing in some specific cases^15^, we further examined the splicing patterns of two edited genes (*EMX1* and *ACTG1*) that are expressed in 293FT cells. Reverse-transcription PCR demonstrated that the edited exons were spliced correctly with the upstream and downstream exons (Supplementary Fig. 6a–d), suggesting that no aberrant splicing events were triggered. We also compared spegRNAs with regular pegRNAs for generating single-base editing, pathogenic point mutations, or repairing preinstalled mutations in other human cells (e.g., U2OS and HeLa) and found that optimized spegRNAs induced higher editing efficiencies than regular pegRNAs in these two cell lines (Supplementary Fig. 7a–i).

Although some optimized spegRNAs could significantly increase the prime editing efficiency, not all of them were found to have beneficial effects (Fig. 1a and Supplementary Figs. 1 and 2). Thus, we sought to explore the rule to design spegRNAs with high efficiency. We first analyzed the relationship between additional base substitution numbers and editing efficiency and discovered that introducing no more than four additional base substitutions in RTT could significantly improve the editing efficiency (Fig. 1b) and that introducing two additional base substitutions induced the highest efficiencies (*P* = 7.4 × 10^−10^, Wilcoxon one-tailed signed-rank test, Fig. 1b). Furthermore, we examined how the position of additional base substitutions could affect the editing efficiency. To characterize the effect of position more comprehensively, we introduced single additional base substitution at positions 1 to 9 in RTT (3′-end to 5′-end, counting the 3′-base of RTT as position 1) at four tested target sites (*ACE2*, *EMX1*, *SITE3*, and *RUNX1*, Supplementary Fig. 8a). After examining the data from all 114 single additional base substitution-containing pegRNAs across thirteen target sites (Fig. 1c), we found that the introduction of single additional base substitution at positions 1, 2, 3, 5, and 6 significantly improved the intended base editing efficiency (median 1.28-, 1.41-, 1.23-, 1.62-, and 1.32-fold, respectively, Fig. 1d), while adding mutations at positions 4, 7, 8, and 9 did not significantly increase the editing efficiency (median 1.01-, 0.92-, 0.93-, and 0.65-fold, respectively, Fig. 1d). As two additional base substitutions induced the highest editing efficiencies in pilot assays (Fig. 1b), we then tested the effects of dual additional base substitutions at positions 1/4, 2/5, and 3/6 (Supplementary Fig. 8b), which were set to generate SSMs in the same open reading frame (ORF). Statistical analyses of the data from all 38 dual additional base substitution-containing pegRNAs across nine target sites (Fig. 1e) showed that adding dual additional base substitutions at positions 1/4, 2/5, and 3/6 could significantly enhance the intended editing frequencies (median 1.20-, 1.90-, and 1.41-fold, respectively, Fig. 1f). Although adding dual additional base substitutions induced even higher editing efficiency than adding single substitution for positions 2/5 and 3/6, adding dual substitutions at position 1/4 induced a lower efficiency than that acquired by adding single substitution at position 1, consistent to the result that adding single substitution at position 4 did not improve editing efficiency (Fig. 1d). Similar results were also observed in previous studies^9,16^.

In addition, we examined whether the type of additional base substitution in RTT affects the editing efficiency. Eleven of twelve tested types of additional base substitutions (e.g., transitions or transversions) in RTT did not generally alter the intended editing efficiency, with only one transversion triggered decreased efficiency (Supplementary Fig. 9a–c). This result suggested that the type of additional base substitution did not significantly affect editing efficiency. Then, we tested whether the length of primer-binding site (PBS) could affect the effect of spegRNAs and found that spegRNAs with different lengths of PBS all generated higher levels of intended single-base editing than the corresponding regular pegRNAs (Supplementary Fig. 9d), without changing the unintended indel frequencies (Supplementary Fig. 9e). These results suggested that the length of PBS did not influence the effect of adding additional base substitution.

Based on these phenomena, we suggest introducing SSMs at five positions (1, 5, 6, 2/5, and 3/6, counting the 3′-base of RTT as position 1, Fig. 1g) when designing spegRNAs. Therefore, at least one or two spegRNAs (positions 6 and 3/6 in ORF1, 1 in ORF2, 5 and 2/5 in ORF3, Fig. 1g) can be designed, and no more than five spegRNAs are required to be constructed in the cases that SSMs can be introduced into the first or second position of some triplet codons. To further test the robustness of the above-generalized rules for spegRNA design (Fig. 1g), we applied the rule to design six spegRNAs against five new target sites (Supplementary Fig. 10a). We found that all spegRNAs designed following our rule (Fig. 1g) induced highly efficient editing at these sites (up-to 81.7%, Supplementary Fig. 10a), at which regular pegRNAs barely induced observable editing (<3%, Supplementary Fig. 10a). After analyzing the results from all target sites tested in this study (Supplementary Fig. 10a, b), we discovered that the spegRNAs designed according to the rule shown in Fig. 1g induced significantly higher editing frequencies than regular pegRNAs (on-average 353-fold, Fig. 1h), though spegRNAs also triggered slightly higher unintended indel frequencies (on-average 1.4-fold Fig. 1i).

### Altered pegRNA secondary structure improves indel efficiency

**Fig. 2:**
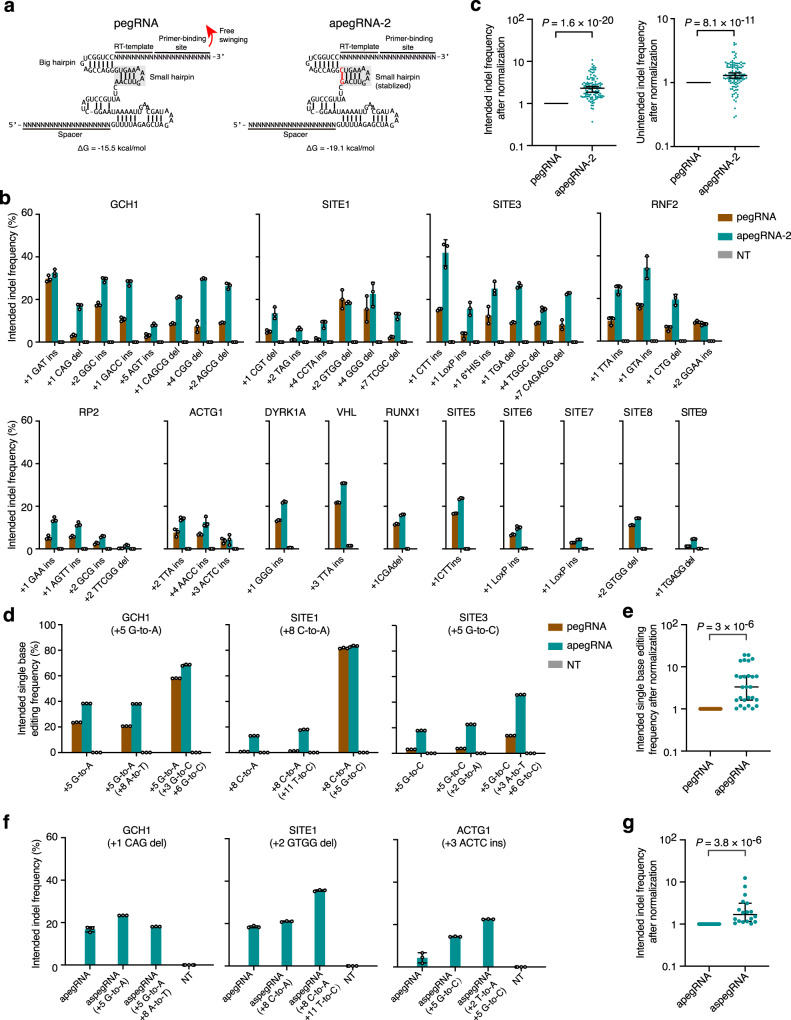
pegRNA with a stabilized secondary structure induced higher efficiencies of intended indel and single-base editing. **a** Schematic diagrams illustrating the predicted secondary structures of regular pegRNA and apegRNA-2. Presumably, the free swinging of the RTT and PBS can break up the small hairpin (left panel), which destabilizes pegRNA. However, engineering within the small hairpin of pegRNAs can stabilize the secondary structures of apegRNA-2 (right panel). **b** Intended indel frequencies were induced by pegRNA and apegRNA-2 at the indicated target sites under the PE3 setting or from non-transfected (NT) cells. **c** Statistical analyses of the intended indel frequencies and unintended indel frequencies after normalization, setting the frequencies induced by regular pegRNAs as 1. *n* = 117 editing from three independent experiments shown in **b**. **d** Intended single-base editing frequencies were induced by pegRNA and apegRNA without or with additional base substitutions at the indicated target sites under the PE3 setting. **e** Statistical analysis of normalized single base editing frequencies, setting the frequencies induced by regular pegRNAs as 1. *n* = 18 editing from three independent experiments shown in **d**. **f** Intended indel frequencies were induced by apegRNA and aspegRNA (with additional base substitutions) at the indicated target sites under the PE3 setting. **g** Statistical analysis of normalized intended indel frequencies, setting the frequencies induced by apegRNA without additional base substitution as 1. *n* = 18 editing from three independent experiments shown in **f**. **b**, **d**, **f** Means ± s.d. are from three independent experiments. **c**, **e**, **g** *P* value, Wilcoxon one-tailed signed-rank test. The median and interquartile range (IQR) are shown. Source data are provided as a Source Data file.

Another application of PE is to introduce small indels^1–3^. As small indels can be readily resolved by endogenous MMR^10^, we sought to use an alternative strategy to enhance the indel-editing efficiency of PE3. Compared to canonical sgRNA, pegRNA contains two extra parts, i.e., PBS and RTT, at its 3′-end. We assumed that the small hairpin of regular pegRNA could be broken up by the free swinging of PBS and RTT, thus compromising the secondary structure stability of pegRNA (Fig. 2a, left panel). Therefore, we altered the backbone of pegRNA to stabilize its secondary structure by inserting a C/G pair (apegRNA-1) or changing each non-C/G pair to a C/G pair (apegRNA-2, -3, -4, -5, Supplementary Fig. 11a) in the small hairpin of pegRNA. We observed that apegRNA-2, which has a C/G pair at the bottom of the small hairpin, induced intended indel editing with higher efficiency than the regular pegRNA and other apegRNAs (Supplementary Fig. 11b). Furthermore, we changed more A/U pairs to C/G pairs in the small hairpin or engineered pegRNA according to a previously published method^17^ (Supplementary Fig. 11c), but the intended indel frequencies induced by the corresponding apegRNAs were not significantly different from those induced by regular pegRNAs (Supplementary Fig. 11d).

We further compared the intended indel efficiencies induced by the regular pegRNA with those induced by apegRNA-2 for generating 39 types of small indels across 14 on-target sites in 293FT cells (Fig. 2a, b). Compared to the regular pegRNA, apegRNA-2 significantly improved the intended indel efficiency in PE3 system (Fig. 2c), with a maximal improvement up-to 10.6-fold (on-average 2.77-fold). We also examined the unintended indel frequencies (Supplementary Fig. 12) and byproducts (Supplementary Fig. 13) at on-target sites and the indel frequencies at predicted pegRNA-dependent OT sites (Supplementary Fig. 14a). We found that although apegRNA-2 rarely triggered byproducts (Supplementary Fig. 13) or OT indels (Supplementary Fig. 14b), it induced higher unintended indel frequencies at on-target sites compared to regular pegRNAs (on-average 1.44-fold, Fig. 2c). To further examine the efficacy of apegRNA in other cells, we compared the editing frequencies of regular pegRNAs and apegRNAs by inducing five types of indels across three target sites in U2OS cells and found that apegRNA-2 significantly improved indel-editing efficiencies (Supplementary Fig. 15a–d). We also tested whether the apegRNA-2 design could be used to improve the editing efficiency of the canonical sgRNA-Cas9 system and found that sgRNA with a small hairpin same to apegRNA-2 (asgRNA) induced indel frequencies similar to those of regular sgRNA at two tested sites as well (Supplementary Fig. 16a–c). Interestingly, higher indel frequencies were induced by asgRNA at one tested site, suggesting that stabilizing the hairpin could prevent sgRNA backbone destabilization (Supplementary Fig. 16a–c). In addition, we also tested the effect of PBS length on the editing efficiencies of the regular pegRNA and apegRNA-2 and found that apegRNA-2 induced higher efficiencies than the regular pegRNA with different lengths of PBS (Supplementary Fig. 16d). Therefore, we selected apegRNA-2 as the apegRNA used in the rest of this study.

As the spegRNA and apegRNA strategies engineer different parts of pegRNA (i.e., RTT and small hairpin), we tested whether the combination of these two strategies can further improve the editing efficiency. The use of apegRNA can induce significantly higher intended single-base editing than the regular pegRNA (Fig. 2d, e) and the inclusion of additional base substitutions into apegRNA also enhanced the intended single-base editing efficiency (Fig. 2d). Moreover, when apegRNA was used to induce the intended small indels, the introduction of certain additional base substitutions (aspegRNA) could further improve the intended indel frequency (Fig. 2f, g). These results suggested that spegRNA and apegRNA could be combined to boost intended single-base editing or indel editing. In addition, we also tested whether spegRNA and apegRNA can be adopted into the PE2 system, in which nicking sgRNA is not required, and we found that both spegRNA and apegRNA improved the editing efficiency of PE2 (Supplementary Fig. 17a–d).

### Comparison and combination of PE5, spegRNA, and apegRNA

**Fig. 3:**
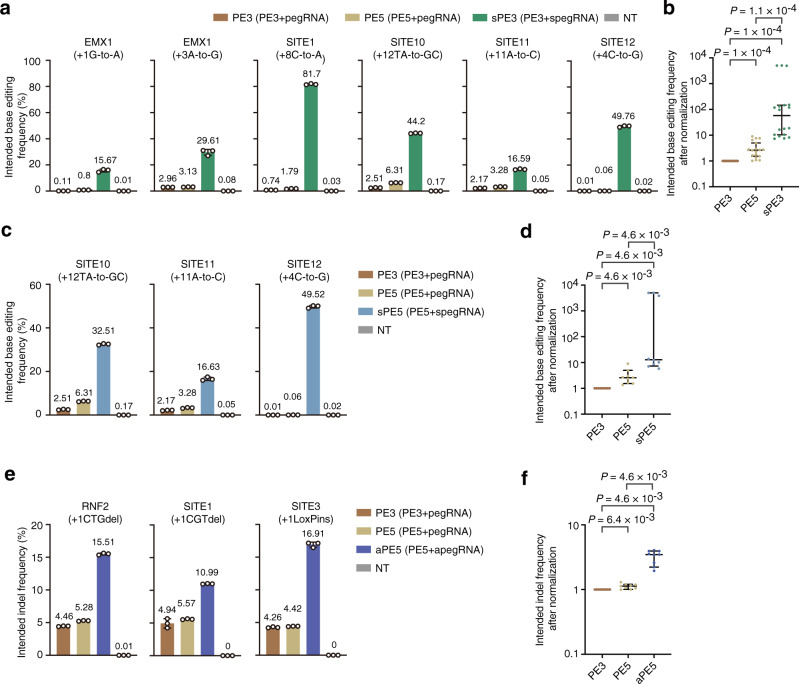
Comparison and combination of PE5, spegRNA, and apegRNA. **a** Comparison of PE5 with spegRNA. The intended base editing frequencies were induced by PE3, PE5, and sPE3 (PE3 with spegRNA) at the indicated target sites. **b** Statistical analysis of normalized base editing frequencies induced by PE3, PE5, and sPE3, setting the frequencies induced by PE3 as 1. *n* = 18 intended base editing from three independent experiments in **a**. **c** Combination of PE5 and spegRNA. The intended base editing frequencies were induced by PE3, PE5, and sPE5 (PE5 with spegRNA) at the indicated target sites. The data for PE3 and PE5 are same as the ones in **a**. **d** Statistical analysis of normalized base editing frequencies induced by PE3, PE5, and sPE5, setting the frequencies induced by PE3 as 1. *n* = 9 intended base editing from three independent experiments in **c**. **e** Combination of PE5 and apegRNA. The intended indel frequencies were induced by PE3, PE5, and aPE5 (PE5 with apegRNA) at the indicated target sites. **f** Statistical analysis of normalized intended indel frequencies induced by PE3, PE5, and aPE5, setting the frequencies induced by PE3 as 1. *n* = 9 intended indel editing from three independent experiments in **e**. **a**, **c**, **e** Means ± s.d. are from three independent experiments. **b**, **d**, **f** *P* value, Wilcoxon one-tailed signed-rank test. The median and interquartile range (IQR) are shown. Source data are provided as a Source Data file.

Recently, a new PE system (PE5) was developed by co-expressing a free dominant-negative mismatch repair protein (MLH1dn) to improve prime editing efficiency^9^. Then we compared the PE3, sPE3 (PE3 with spegRNA) and PE5 systems for generating six types of edits across five target sites and found that PE5 induced on-average 3.42-fold increase (maximal 7.27-fold) compared to PE3 (Fig. 3a, b). Dramatically, sPE3 induced on-average 877-fold increase (maximal 4976-fold, from 0.01% to 49.76%, SITE12) compared to PE3 (Fig. 3a, b) at these sites. Next, we also examined whether spegRNA and apegRNA can be combined with PE5 to further enhance editing efficiency and found that both sPE5 (PE5 with spegRNA) and aPE5 (PE5 with apegRNA) can induced even higher editing efficiency than PE5 (Fig. 3c–f).

## Discussion

As a versatile editing tool with high product purity^1–3,8,18^ and editing specificity^4–6^, PE has great potential in the application of correcting pathogenic mutations to treat genetic disorders^16,19–21^. Although PE3 induced efficient editing at some target sites, its efficiency remained generally low at many target sites, including those associated with human diseases (Fig. 1a and Supplementary Fig. 2b, f and previous studies^8^). Here, we developed two strategies, spegRNA by introducing SSMs at the proper positions of RTT or apegRNA by stabilizing the RNA secondary structure, to enhance the editing efficiency of PE3 to generate single-base substitutions or indels across multiple target sites in various human cells. During the revision of this manuscript, other studies also found that adding additional base substitution into the RTT of pegRNA can increase prime editing efficiency in plants but the detailed rule for designing such pegRNA was not revealed yet^22^, which may lead to the generation of pegRNAs with no improved efficiency or even decreased efficiency in human cells (Fig. 1a and Supplementary Figs. 1 and 2). After analyzing the efficiencies of 152 types of edits across 13 target sites (Fig. 1c–f and Supplementary Fig. 8), we summarized a rule of spegRNA design (Fig. 1g). According to our rule, highly efficient editing can be achieved in human cells (Fig. 3a), by testing no more than 5 types of spegRNAs (Fig. 1g). As different parts of pegRNA were engineered in spegRNA and apegRNA, they can be combined to further improve PE’s editing efficiency (aspegRNA, Fig. 2d–g). Moreover, both spegRNA and apegRNA can be applied in the recently reported PE5 system^3^ to even increase its efficiency (sPE5 and aPE5, Fig. 3c–f). Theoretically, the spegRNA and apegRNA strategy should also be compatible with other recently reported methods^5,23,24^ to even increase editing efficiency, which awaits further investigation. Different from other improved PE systems^5,23,24^, neither sPE or aPE system requires extra protein or RNA components and thus the total sizes of sPE and aPE are constrained, which facilitates their in vivo delivery (such as viral delivery^25,26^) for the therapeutical applications in the future.

## Methods

### Plasmid construction

The primer set (pegRNA_F/pegSITE3_R) was used to amplify the pegRNA-scaffold-fragment with template pGL3-U6-sgRNA-PGK-puromycin (addgene, 51133). Then the amplified pegRNA-scaffold-fragment was cloned into the BsaI and EcoRI linearized pGL3-U6-sgRNA-PGK-puromycin with NovoRec® plus One step PCR Cloning Kit (NR005, Novoprotein) to generate the vector pGL3-U6-pegRNA-PGK-puromycin for the expression of pegRNA.

Oligonucleotides CXCR4_FOR/CXCR4_REV were annealed and ligated into BsaI linearized pGL3-U6-pegRNA-PGK-puromycin to generate the vector psgCXCR4-spacer. Oligonucleotides CXCR4_5_FOR/CXCR4_5_REV were annealed and ligated into the PflFI and EcoRI linearized psgCXCR4-spacer to generate the vector ppegCXCR4 + 5G-to-T for the expression of pegCXCR4 + 5G-to-T. Other expression vectors for pegRNA and spegRNA were constructed by the similar strategy.

Oligonucleotides CXCR4_nick_FOR/CXCR4_nick_REV were annealed and ligated into BsaI linearized pGL3-U6-sgRNA-PGK-puromycin to generate the vector pnick-sgCXCR4 for the expression of nick-sgCXCR4. Other expression vectors for nick-sgRNA were constructed by the similar strategy.

The primer set (pegRNA_2024plusGC_F/pegRNA_2024plusGC_R) was used to insert a G/C pair in pGL3-U6-pegRNA-PGK-puromycin and generate pGL3-U6-apegRNA1-PGK-puromycin. The primer set (pegRNA_1629CG_F/pegRNA_1629CG_R) was used to change a G/A mismatch to a C/G pair in pGL3-U6-pegRNA-PGK-puromycin and generate pGL3-U6-apegRNA2-PGK-puromycin. Other expression vectors for apegRNA were constructed by the similar strategy.

Oligonucleotides GCH1_FOR/GCH1_REV were annealed and ligated into BsaI linearized pGL3-U6-apegRNA1-PGK-puromycin and pGL3-U6-apegRNA2-PGK-puromycin to generate the vector psgGCH1-spacer-1 and psgGCH1-spacer-2. Oligonucleotides pegGCH1_+1GATins_FOR/ pegGCH1_+1GATins_REV were annealed and ligated into the PflFI and EcoRI linearized psgGCH1-spacer-1 and psgGCH1-spacer-2 to generate the vector papegGCH1_+1GATins-1 and papegGCH1_+1GATins-2 for the expression of apegGCH1_+1GATins-1 and apegGCH1_+1GATins-2. Other expression vectors for apegRNA were constructed by the similar strategy.

The sequences of the oligos used for plasmid construction are listed in Supplementary Data 1.

### Cell culture and transfection

293FT (Thermo Fisher Scientific, R70007), U2OS (ATCC^®^ HTB-96) and HeLa (ATCC^®^ CCL-2™) cells were maintained in DMEM (10566, Gibco/Thermo Fisher Scientific) + 10% FBS (16000-044, Gibco/Thermo Fisher Scientific) and regularly tested to exclude mycoplasma contamination.

For prime editing with pegRNA (spegRNA or apegRNA), 293FT, U2OS or Hela cells were seeded in a 24-well plate at a density of 1×10^5^ per well and transfected with 250 μl serum-free Opti-MEM that contained 2.6 μl LIPOFECTAMINE LTX (Life, Invitrogen), 1.3 μl LIPOFECTAMINE plus (Life, Invitrogen), 0.9 μg PE2 expression vector, 0.3 μg pegRNA (spegRNA or apegRNA) expression vector with 0.1 μg nick-sgRNA expression vector. After 24 h, puromycin (ant-pr-1, InvivoGen) was added to the medium at the final concentration of 4 μg/ml. After another 48 h, the genomic DNA was extracted from the cells with QuickExtract^TM^ DNA Extraction Solution (QE09050, Epicentre) for subsequent sequencing analysis.

### Cell line construction

To establish ACE2-S19A cell line, the 293FT cells were seeded into a 60-mm plate at a density of 4 × 10^5^ per well and cultured for 24 h. Cells were transfected with plasmids expressing PE2, pegACE2-S19A and nick-sgACE2, according to the manufacturer’s instruction. After 48 h, 10 μg/ml puromycin was added into the media for two days. ACE2-S19A cell line expanded from a single-clone and was validated by genomic DNA sanger sequencing. HBB-E7V cell line was constructed by the similar strategy. PGM3-L111S cell line was constructed as previously reported^27^. Briefly, to generate the pathogenic mutation at *PGM3* loci, 293FT cells were seeded into a six-well plate and transfected with ABEmax and the corresponding sgRNA-expressing plasmid. The genomic DNAs of single-cell clones were individually purified, and the clone containing intended pathogenic mutation was validated by Sanger sequencing.

### DNA library preparation and sequencing

Target genomic sequences were PCR amplified by Phanta® Max Super-Fidelity DNA Polymerase (P505, Vazyme) with primer sets flanking examined pegRNA target sites. The pegRNA target sequences and PCR primers were listed in Supplementary Data 2. Indexed DNA libraries were prepared by using the NEBNext Ultra II FS DNA Library Prep Kit for Illumina. After quantitated with Qubit High-Sensitivity DNA kit (Invitrogen), PCR products with different tags were pooled together for deep sequencing by using the Illumina HiSeq X10 (2 × 150) or NovaSeq 6000 (2 × 150) at Shanghai Institute Nutrition and Health, Big Data Center Omics Core, Shanghai, China. Raw read qualities were evaluated by FastQC (v0.11.8, http://www.bioinformatics.babraham.ac.uk/projects/fastqc/). For paired-end sequencing, only R1 reads were used. Adaptor sequences and read sequences on both ends with Phred quality score lower than 30 were trimmed. Clean reads were then mapped with the BWA-MEM algorithm (v0.7.17-r1188) to target sequences. After piled up with Samtools (v1.9), editing frequencies were further calculated according to previously published literatures^28^.

### Base substitution frequency calculation

Base substitutions were selected at each base of the examined pegRNA target sites that were mapped with at least 1,000 independent reads, and obvious base substitutions were only observed at the targeted base editing sites. Base substitution frequencies were calculated by dividing base substitution reads (without indels) by total reads using CFBI pipeline (https://github.com/YangLab/CFBI, v1.0.0)^28^. Counts of reads for individual bases at examined target sites and pegRNA-dependent OT sites are listed in Supplementary Data 3 and 5, respectively.

### Indel frequency calculation at on-target sites

Intended indel refers to the insertion/deletion designed in pegRNAs. Unintended indel refers to undesired editing outcome containing indel around nCas9 cleavage site. Intended indel frequencies were calculated as (count of reads with only intended indel at the target site)/(count of total reads covering the target site). These counts are listed in Supplementary Data 4. Unintended indel frequencies were estimated among reads aligned in the region spanning from upstream 8 nucleotides to the target site to downstream 52 nucleotides to PAM site (80 bp). Unintended indel frequencies for base substitution were calculated according to reported CFBI pipeline (https://github.com/YangLab/CFBI, v1.0.0)^28^ as (count of reads containing at least one unintended inserted and/or deleted nucleotide)/(count of total reads aligned in the estimated region). Unintended indel frequencies for targeted insertion/deletion were calculated as (count of reads containing unintended indels)/(count of total reads aligned in the estimated region). These counts are listed in Supplementary Data 4.

### Incomplete products and byproducts frequencies calculation

Incomplete products refer to the editing outcomes with only additional base substitution but no intended base editing, and byproducts refer to the editing outcomes with pegRNA scaffold incorporation, here. After recalling all mutation types on each read, incomplete products frequencies were calculated as (count of reads only containing incomplete product)/(count of total reads covering the target sites), byproducts frequencies were calculated as (count of reads only containing byproduct)/(count of total reads covering the target sites).

### Indel frequency calculation at pegRNA-dependent OT site

Indel frequencies for pegRNA-dependent OT site insertion/deletion were estimated among reads aligned in the region spanning from upstream 8 nucleotides to OT site to downstream 52 nucleotides to PAM site (80 bp), and calculated according to reported CFBI pipeline (https://github.com/YangLab/CFBI, v1.0.0)^28^ as: (count of reads containing at least one unintended inserted and/or deleted nucleotide)/(count of total reads aligned in the estimated region). These counts are listed in Supplementary Data 5.

### Predication of pegRNA-dependent OT site

Potential pegRNA-dependent OT sites were predicted by Cas-OFFinder^14^, allowing up-to 5 mismatches.

### RNA extraction and reverse transcription

Total RNAs were extracted with the TransZol® Up Plus RNA Kit (TransGen, Beijing, China) and reversely transcribed with cDNA Synthesis SuperMix (TransGen) according to the manufacturer’s instructions. Target cDNA sequences were PCR amplified by Phanta® Max Super-Fidelity DNA Polymerase (P505, Vazyme) with primer sets flanking examined pegRNA target sites. The PCR primers were listed in Supplementary Data 2.

### Predication of RNA secondary structure

Secondary structures of used apegRNAs were predicted by RNAfold^29^.

### Statistics and reproducibility

All statistical analyses were performed with R package 4.1.1 (http://www.R-project.org/). *P* values were calculated from Wilcoxon one-tailed signed-rank test in this study. No statistical method was used to predetermine sample size. No data were excluded from the analyses. The experiments were not randomized. Analysis was performed based on numerical names (without the experimental information of samples).

### Reporting summary

Further information on research design is available in the Nature Research Reporting Summary linked to this article.

## Supplementary information

Supplementary Information

Reporting Summary

Description of Additional Supplementary Files

Supplementary Data 1

Supplementary Data 2

Supplementary Data 3

Supplementary Data 4

Supplementary Data 5

## Data Availability

The deep sequencing data generated in this study can be accessed in Gene Expression Omnibus under the accession code GSE197730 and in National Omics Data Encyclopedia under the accession code OEP003181. The processed data about all base substitution frequencies and indels frequencies are provided in Supplementary Data 3–5. All other data supporting the finding of this study are available from the corresponding author on reasonable request. Source data are provided with this paper.

## Source data

Source Data

## Code availability

High-confident base substitution or indel events were predicted by the CFBI pipeline (https://github.com/YangLab/CFBI, v1.0.0)^28^. The custom Perl and Shell scripts for calculating frequencies of base substitution and indels (CFBI) are available at GitHub (https://github.com/YangLab/CFBI, v1.0.0).
