# Supplementary material for: Highly efficient prime editing by introducing same-sense mutations in pegRNA or stabilizing its structure
Source: Nat Commun. 2022 Mar 29;13:1669. doi: 10.1038/s41467-022-29339-9 (PMC8964725; doi:10.1038/s41467-022-29339-9)
Supplement: Supplementary file 1 — Supplementary Information [file 41467_2022_29339_MOESM1_ESM.pdf]

Supplementary figures and legends

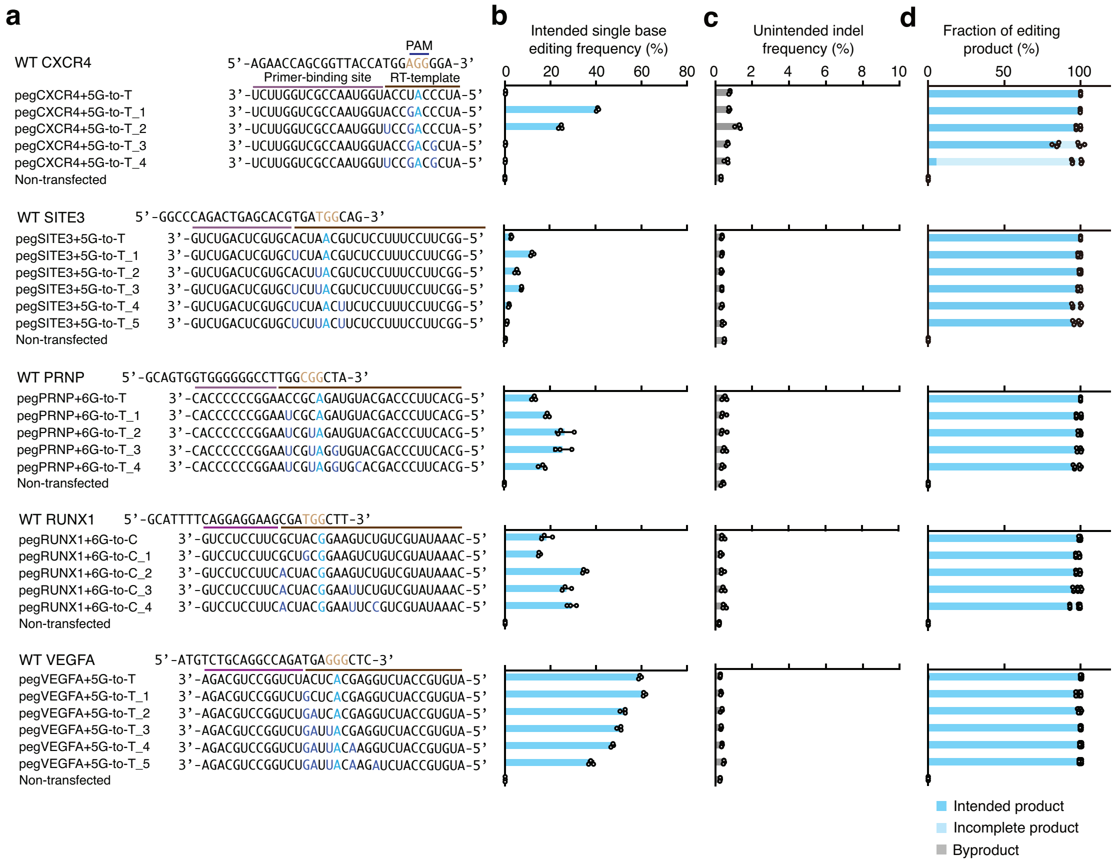

Supplementary Fig. 1. pegRNA containing additional base substitutions induced higher editing efficiencies of single-base substitution at more target sites.

(**a**) Sequences of the PBS and RTT of pegRNAs and WT on-target genomic sites. Intended single-base edits are in cyan, additional base substitutions are in blue and PAMs are in brown. (**b-d**) The intended single-base editing frequencies (**b**), unintended indel frequencies (**c**) and fractions of editing product (**d**) were induced by the pegRNAs in (**a**) at the indicated target sites under the PE3 setting. Means ± s.d. are from three independent experiments. Source data are provided as a Source Data file.

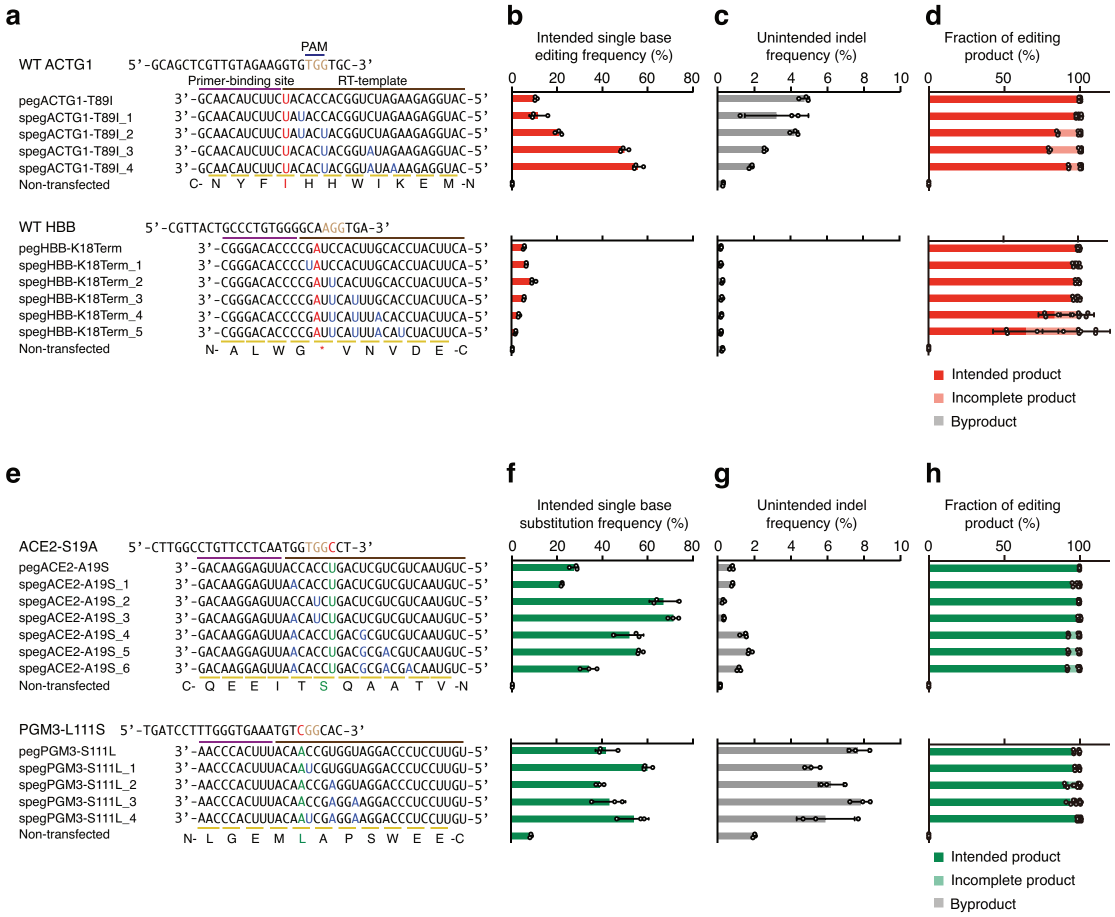

Supplementary Fig. 2. pegRNA containing SSMs induced higher editing efficiencies to generate or repair pathogenic point mutations at more target sites.

(**a**) Sequences of the PBS and RTT of pegRNAs and spegRNAs for generating pathogenic point mutations and WT on-target genomic sites. Pathogenic point mutations are in red and SSMs are in blue. (**b-d**) The intended single-base editing frequencies (**b**), unintended indel frequencies (**c**) and fractions of editing product (**d**) were induced by the indicated pegRNAs and spegRNAs in (**a**). (**e**) Sequences of the PBS and RTT of pegRNAs and spegRNAs for repairing pathogenic mutations and on-target genomic sites with pre-installed mutations. Corrected bases are in green, pre-installed point mutations are in red and SSMs are in blue. (**f-h**) The intended single-

base editing frequencies (**f**), unintended indel frequencies (**g**) and fractions of editing product (**h**) were induced by the indicated pegRNAs and spegRNAs in (**e**). (**b**, **c**, **d**, **f**, **g**, **h**) Means ± s.d. are from three independent experiments. Source data are provided as a Source Data file.

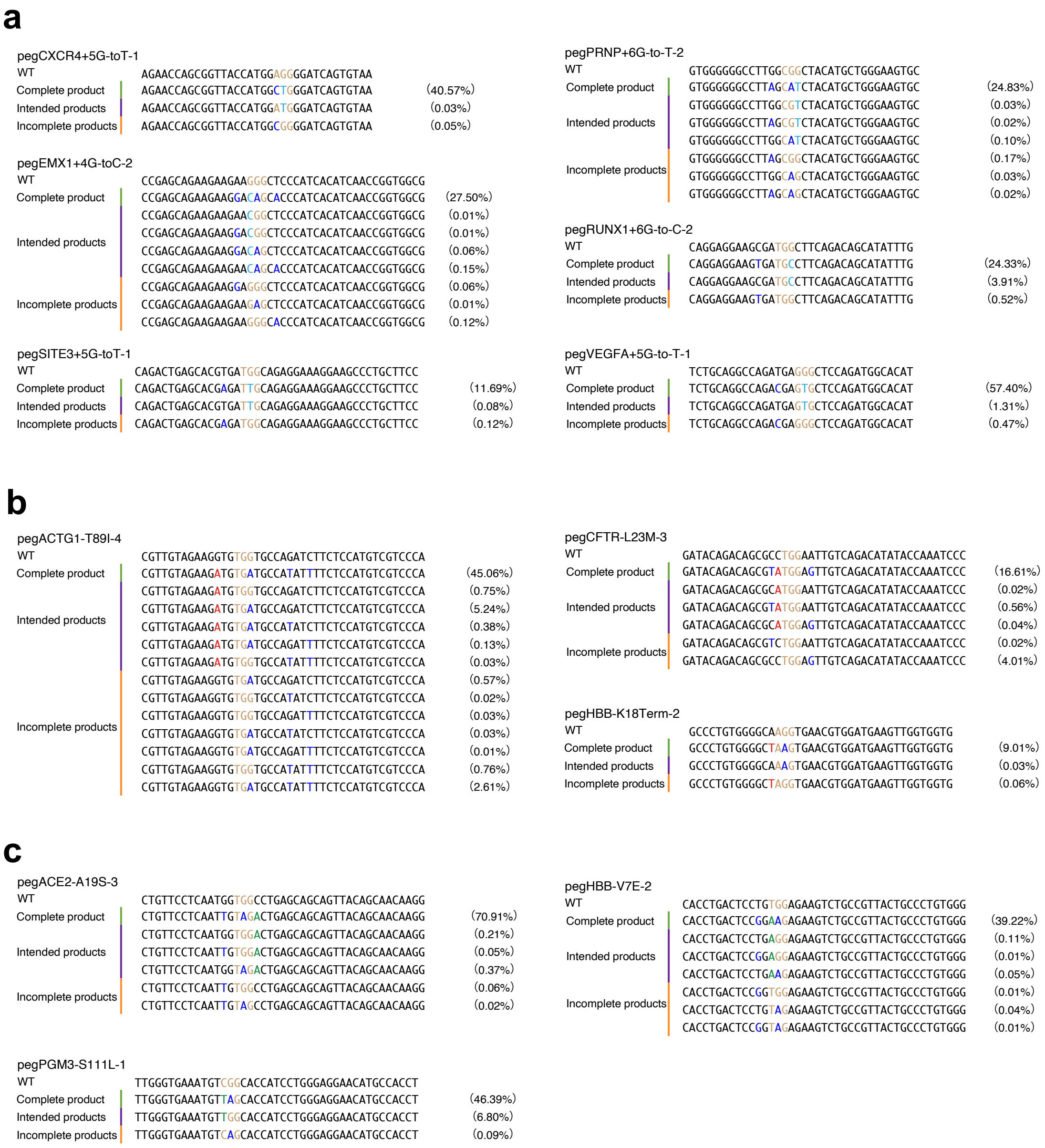

Supplementary Fig. 3. Incomplete products induced by the pegRNAs containing additional base substitutions at on-target sites.

(**a**) The frequencies of complete products (with intended single-base editing and all additional base substitutions), intended products (with intended single-base editing but not all additional base substitutions) and incomplete products (with additional base substitutions but no intended single-base editing) induced by the optimized

pegRNAs with additional base substitutions in Fig. **1a** and Supplementary Fig. **1a**. (**b**) The frequencies of complete products (with intended single-base editing and all SSMs), intended products (with intended single-base editing but not all SSMs) and incomplete products (with SSMs but no intended single-base editing) induced by the optimized spegRNAs in Fig. **1a** and Supplementary Fig. **2a**. (**c**) The frequencies of complete products (with intended single-base editing and all SSMs), intended products (with intended single-base editing but not all SSMs) and incomplete products (with SSMs but no intended single-base editing) induced by the optimized spegRNAs in Fig. **1a** and Supplementary Fig. **2e**. The frequencies are means from three biologically independent experiments.

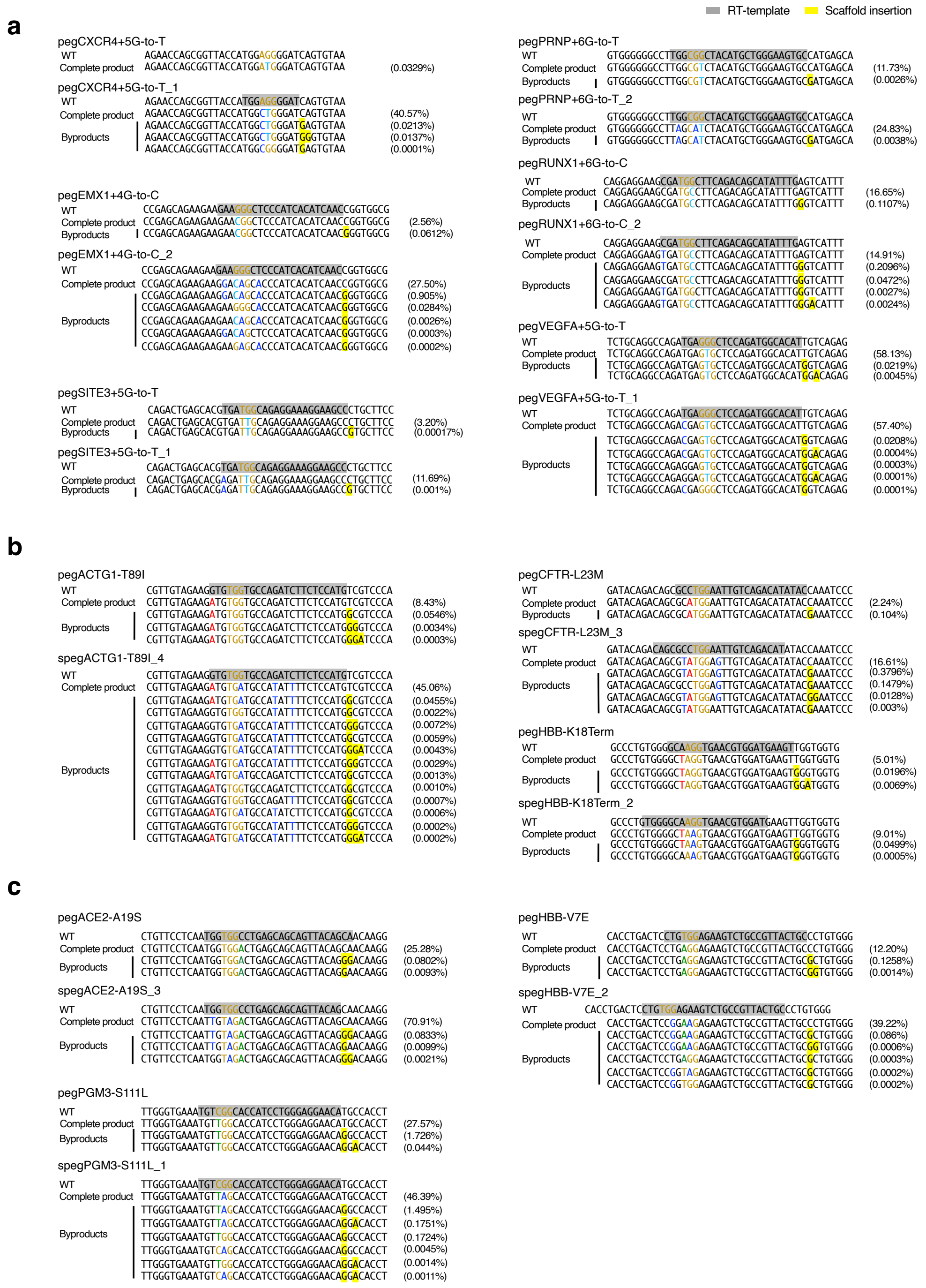

Supplementary Fig. 4. Byproducts induced by the pegRNAs containing additional base substitutions at on-target sites.

(**a**) The frequencies of complete products and byproducts (with pegRNA scaffold incorporation) induced by regular pegRNAs and the optimized pegRNAs containing

additional base substitutions in Fig. **1a** and Supplementary Fig. **1a**. (**b**) The frequencies of complete products and byproducts induced by regular pegRNAs and the optimized spegRNAs in Fig. **1a** and Supplementary Fig. **2a**. (**c**) The frequencies of complete products and byproducts induced by regular pegRNAs and the optimized spegRNAs in Fig. **1a** and Supplementary Fig. **2e**. The frequencies are means from three biologically independent experiments.

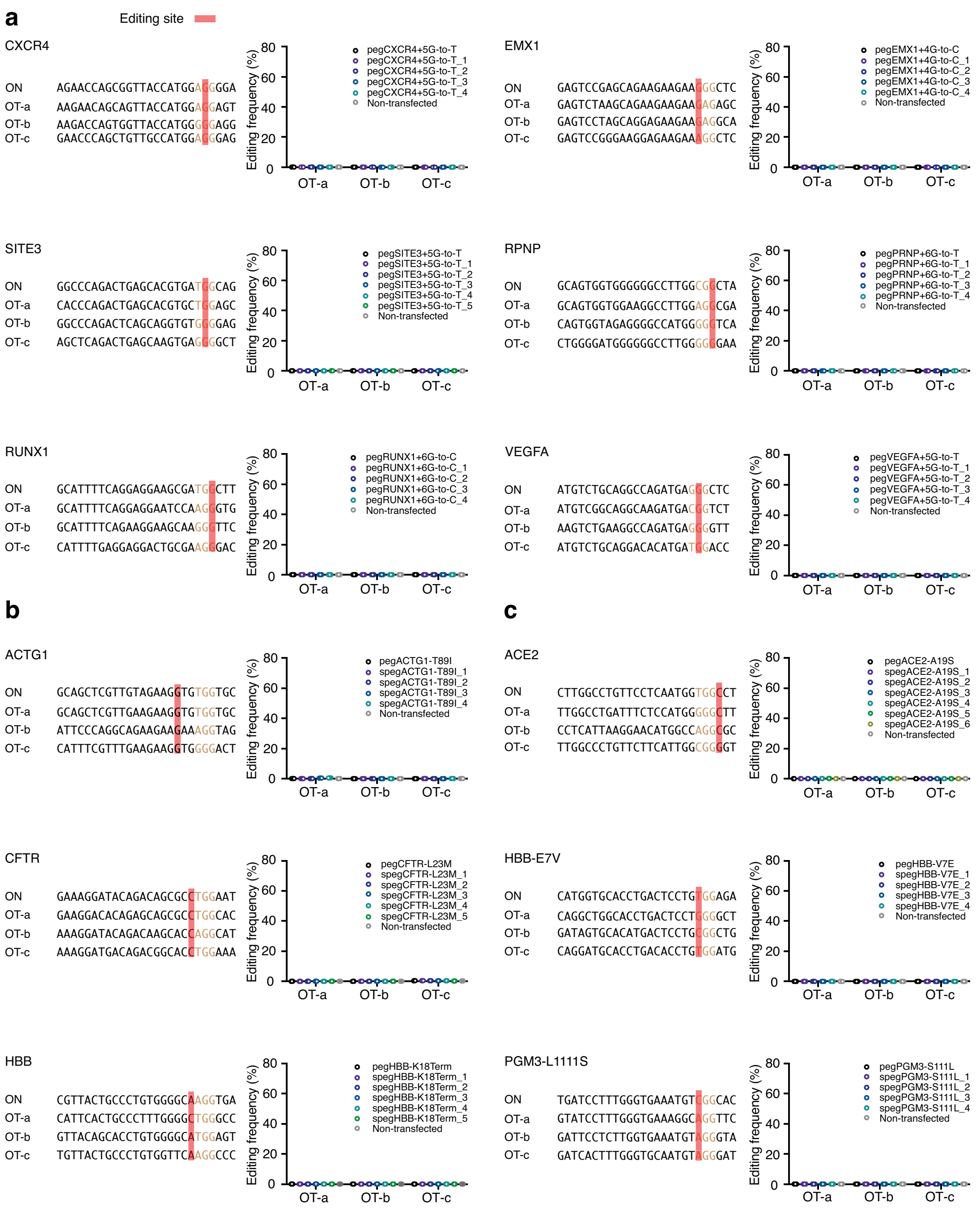

Supplementary Fig. 5. Off-target editing induced by the pegRNAs containing additional base substitutions.

(**a**) The sequences of on- and off-target sites for the pegRNAs in Fig. **1a** and Supplementary Fig. **1a** are shown. The base substitution frequencies were determined at the indicated off-target sites. (**b**) The sequences of on- and off-target sites for the

pegRNAs in Fig. **1a** and Supplementary Fig. **2a** are shown. The base substitution frequencies were determined at the indicated off-target sites. (**c**) The sequences of on- and off-target sites for the pegRNAs in Fig. **1a** and Supplementary Fig. **2e** are shown. The base substitution frequencies were determined at the indicated off-target sites. Means ± s.d. are from three independent experiments. Source data are provided as a Source Data file.

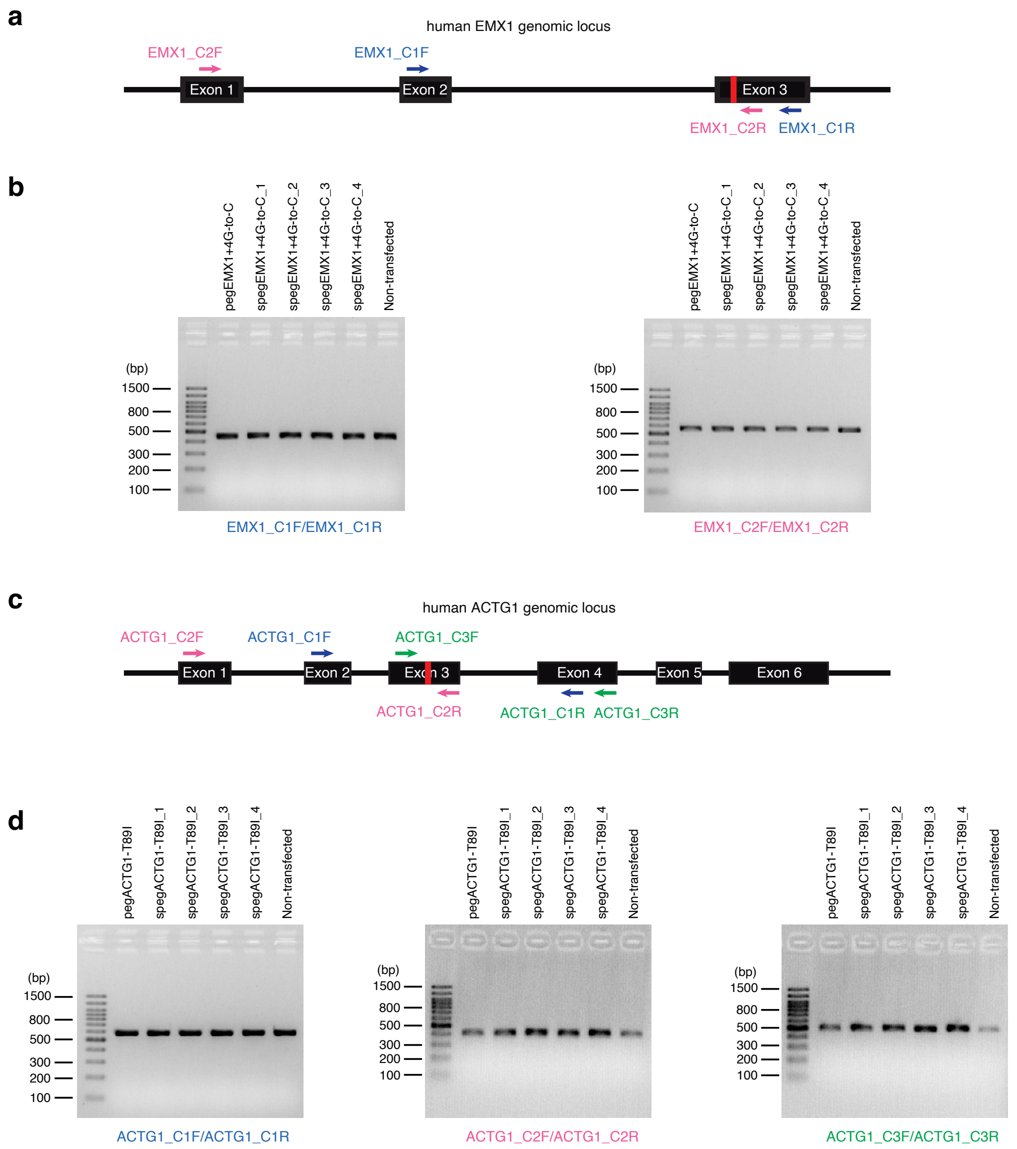

Supplementary Fig. 6. Splicing patterns of the exons edited by the pegRNAs containing additional base substitutions.

(**a**) Schematic diagrams illustrating the position of PCR primers to detect the splicing pattern of the edited exon 3 of *EMX1*. (**b**) RT-PCR results showed that no aberrant splicing of *EMX1* exon 3 was triggered by the indicated pegRNAs. (**c**) Schematic diagrams illustrating the position of PCR primers to detect the splicing pattern of the edited exon 3 of *ACGT1*. (**d**) RT-PCR results showed that no aberrant splicing of

ACGT1 exon 3 was triggered by the indicated pegRNAs. Agarose gel data are representative of three independent experiments. Uncropped gels for (**b**) and (**d**) are provided.

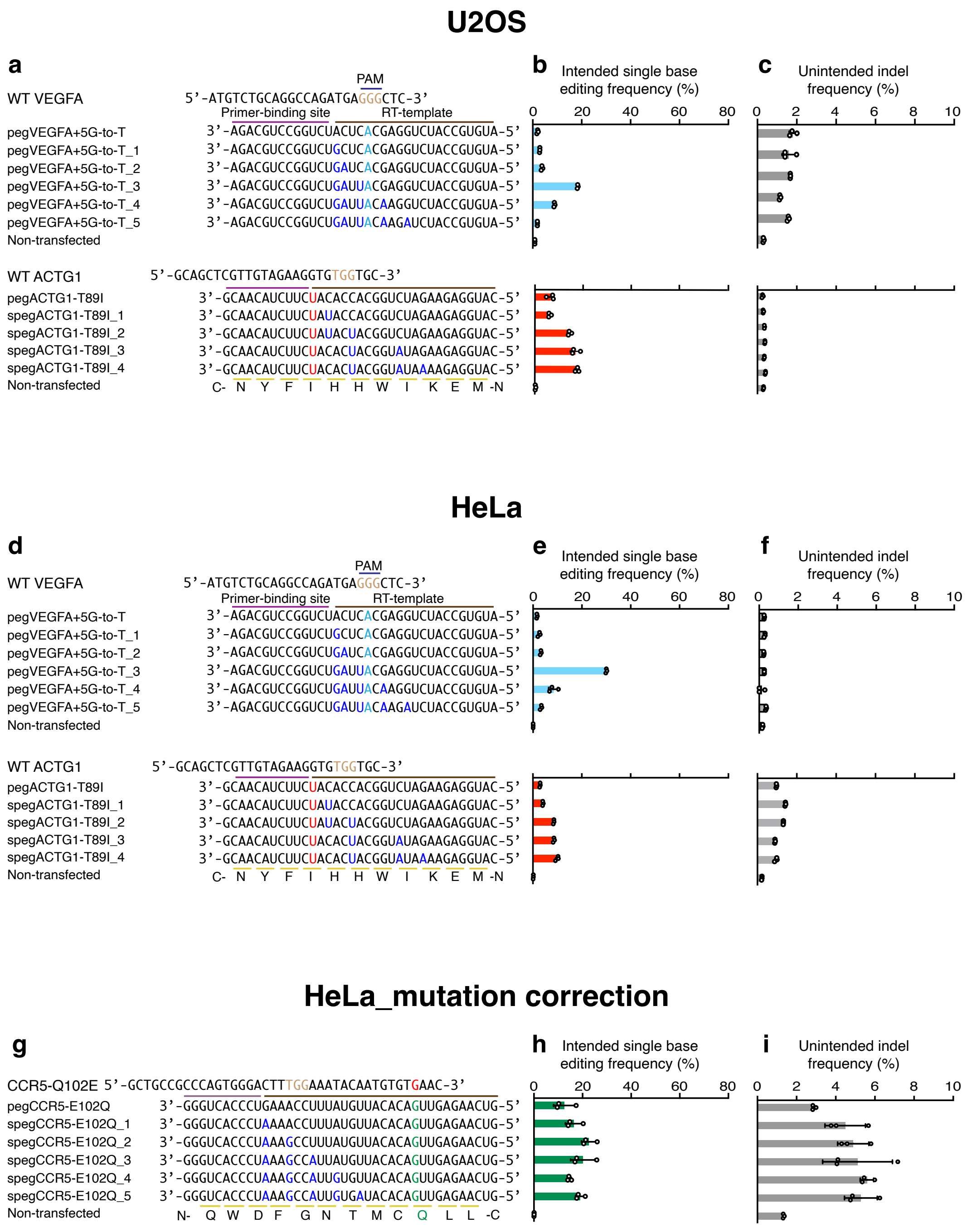

Supplementary Fig. 7. The pegRNA containing additional base substitutions or SSMs induced higher single-base editing efficiencies in different cells.

(**a**) Sequences of the PBS and RTT of pegRNAs and spegRNAs and WT on-target genomic site in U2OS cells. Intended single-base edits are in cyan, pathogenic point mutations are in red and additional base substitutions and SSMs are in blue. (**b**, **c**) The

intended single-base editing frequencies (**b**) and unintended indel frequencies (**c**) were induced by the indicated pegRNAs and spegRNAs in (**a**) in U2OS cells. (**d**) Sequences of the PBS and RTT of pegRNAs and spegRNAs and WT on-target genomic site in HeLa cells. Intended single-base edits are in cyan, pathogenic point mutations are in red and additional base substitutions and SSMs are in blue. (**e**, **f**) The intended single-base editing frequencies (**e**) and unintended indel frequencies (**f**) were induced by the indicated pegRNAs and spegRNAs in (**d**) in HeLa cells. (**g**) Sequences of the PBS and RTT of spegRNAs and on-target genomic site in the HeLa cells with a pre-installed point mutation. Corrected bases are in green, pre-installed point mutations are in red and SSMs are in blue. (**h**, **i**) The intended single-base editing frequencies (**h**) and unintended indel frequencies (**i**) were induced by the indicated pegRNAs in (**g**) in the HeLa cells with the pre-installed point mutation. (**b**, **c**, **e**, **f**, **h**, **i**) Means ± s.d. are from three independent experiments. Source data are provided as a Source Data file.

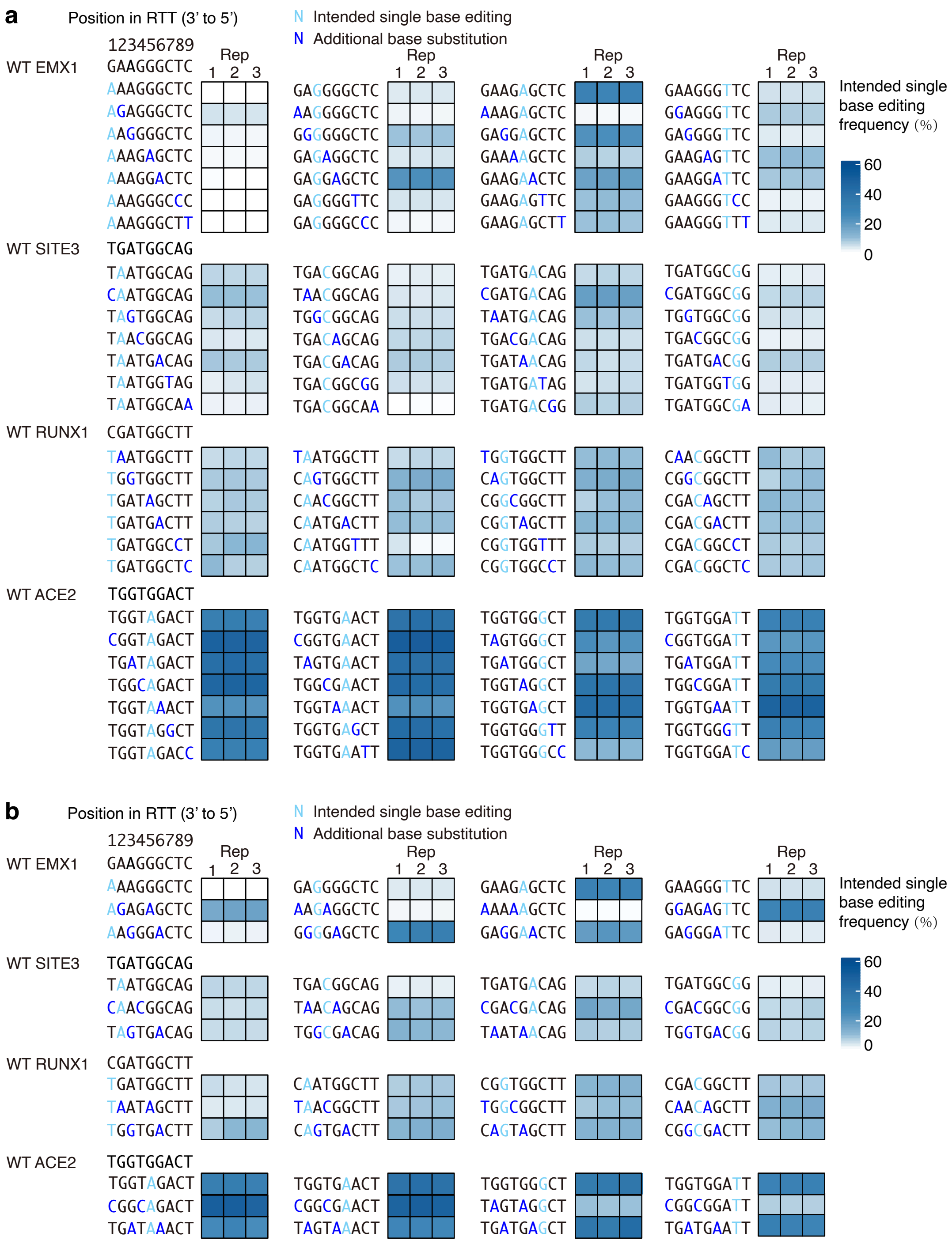

Supplementary Fig. 8. Effect of additional base substitution position in RTT on editing efficiency.

(**a**) Heatmaps show the intended single-base editing frequencies induced by the pegRNAs containing one additional base substitution at the indicated positions in

RTT. (**b**) Heatmaps show the intended single-base editing frequencies induced by the pegRNAs containing two additional base substitution at the indicated positions in RTT. Source data are provided as a Source Data file.

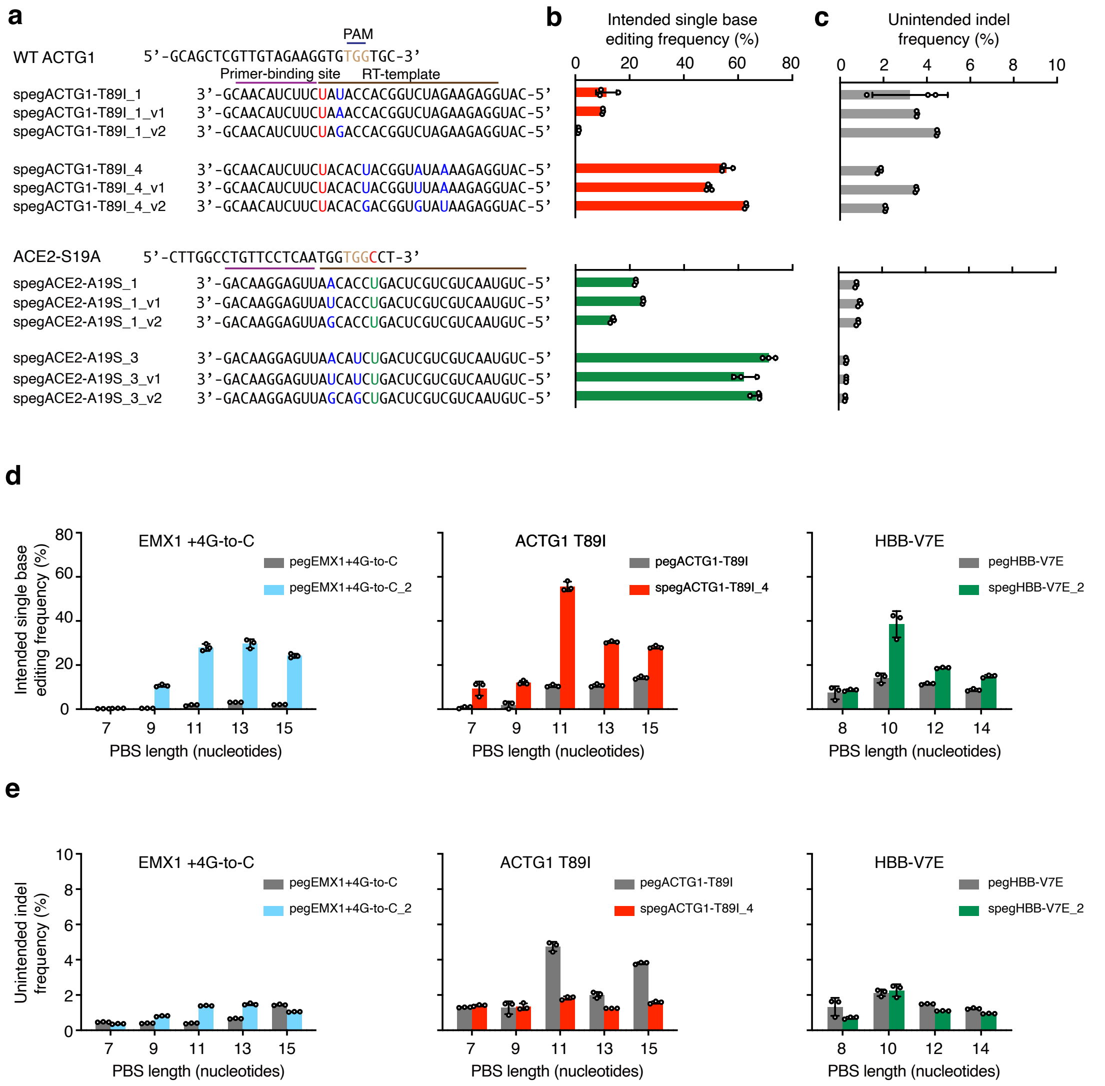

Supplementary Fig. 9. Effects of the mutation type and PBS length of spegRNA on editing efficiency.

(**a**) Sequences of PBS and RTT of the spegRNAs with different kind of SSMs (*e.g.*, transition and transversion). Pathogenic point mutations are in red and SSMs are in blue. (**b**, **c**) The intended single-base editing frequencies (**b**) and unintended indel frequencies (**c**) were induced by the spegRNAs in (**a**). (**d**, **e**) The intended single-base editing frequencies (**d**) and unintended indel frequencies (**e**) induced by the spegRNAs with different length of PBS at the indicated target sites. (**b**, **c**, **d**, **e**) Means

± s.d. are from three independent experiments. Source data are provided as a Source Data file.

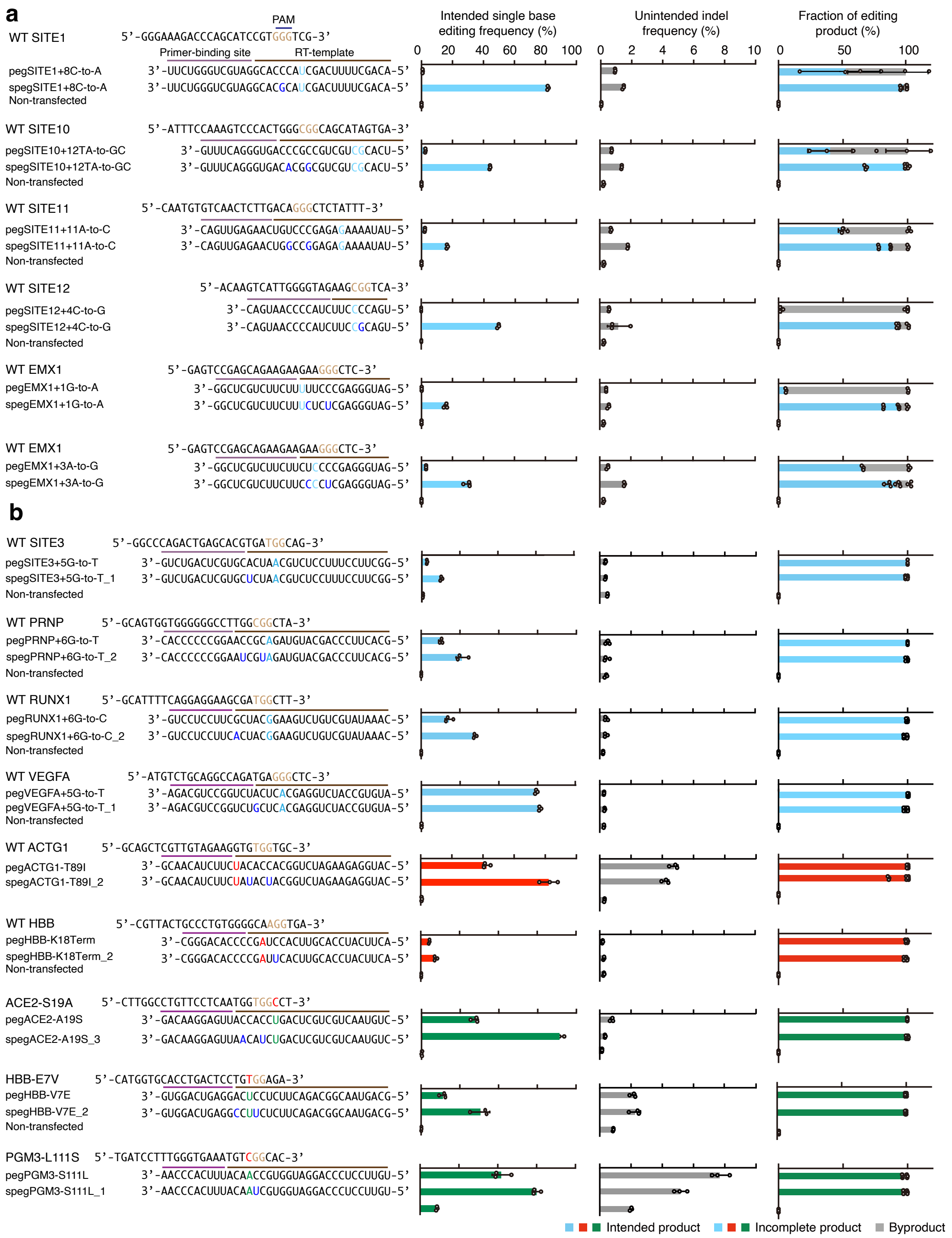

Supplementary Fig. 10. Comparison of the editing efficiencies by pegRNA and spegRNA at more target sites.

(**a**) The intended single-base editing frequencies, unintended indel frequencies and fractions of editing product were induced by the indicated pegRNAs and spegRNAs

at newly tested target sites. (**b**) Reanalyzing the intended single-base editing frequencies, unintended indel frequencies and fractions of editing product that were induced by the indicated pegRNAs and spegRNAs at previously tested target sites. Means ± s.d. are from three independent experiments. The data are from Fig. **1a** and Supplementary Fig. **1** and **2**. (**a, b**). Source data are provided as a Source Data file.

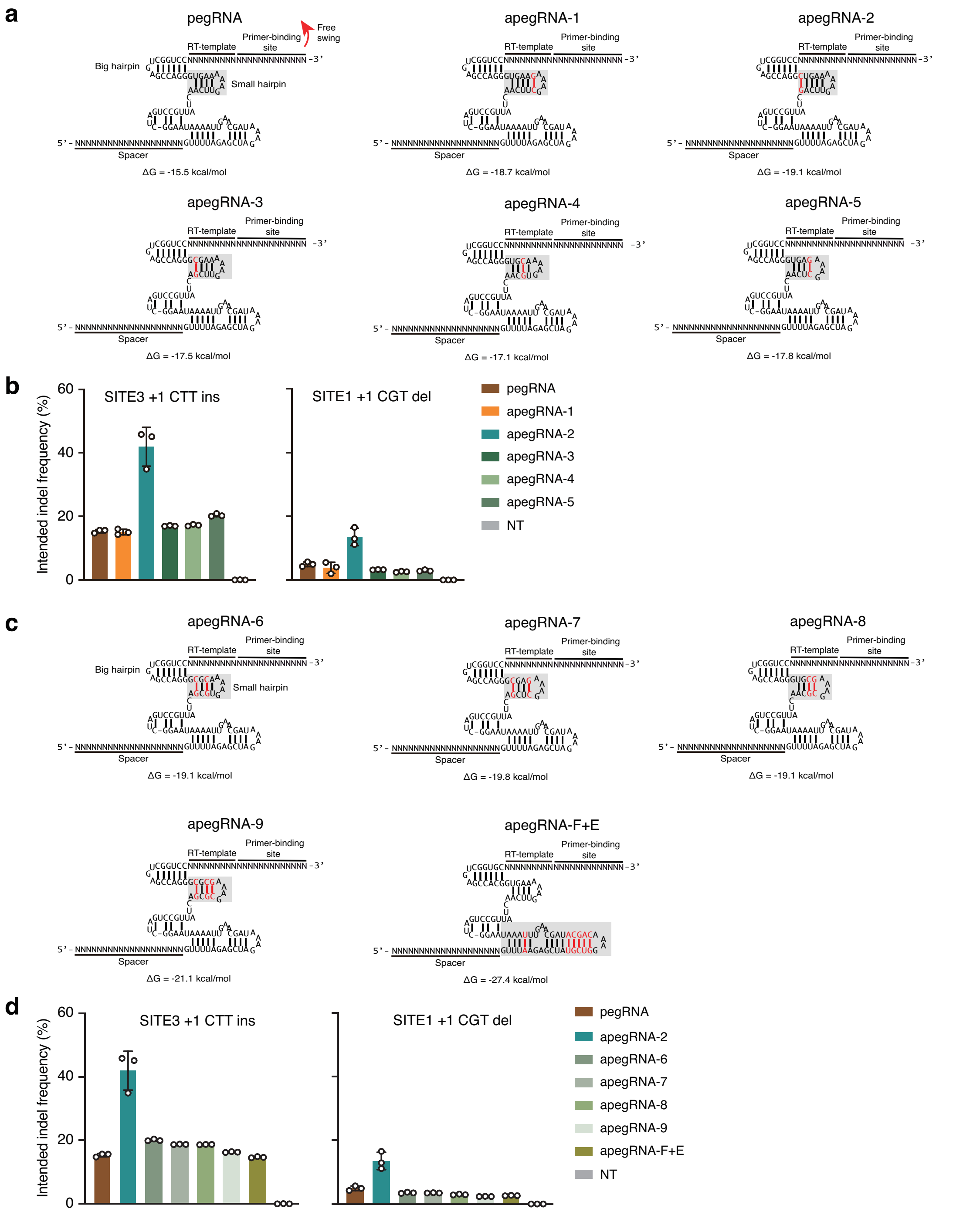

Supplementary Fig. 11. Effect of pegRNA scaffold structure on editing efficiency.

(**a**) Schematic diagrams illustrating the predicted secondary structures of a regular pegRNA and the apegRNAs with one A/U-to-G/C base pair change in the small hairpin. (**b**) Intended indel frequencies were induced by the pegRNAs in (**a**) under the

PE3 setting. (**c**) Schematic diagrams illustrating the predicted secondary structures of the apegRNAs with two A/U-to-G/C base pair change in the small hairpin or with a previously published structure[17]. (**d**) Intended indel frequencies were induced by apegRNA-2 and the pegRNAs in (**c**) under the PE3 setting. (**b**, **d**) Means ± s.d. are from three independent experiments. Source data are provided as a Source Data file.

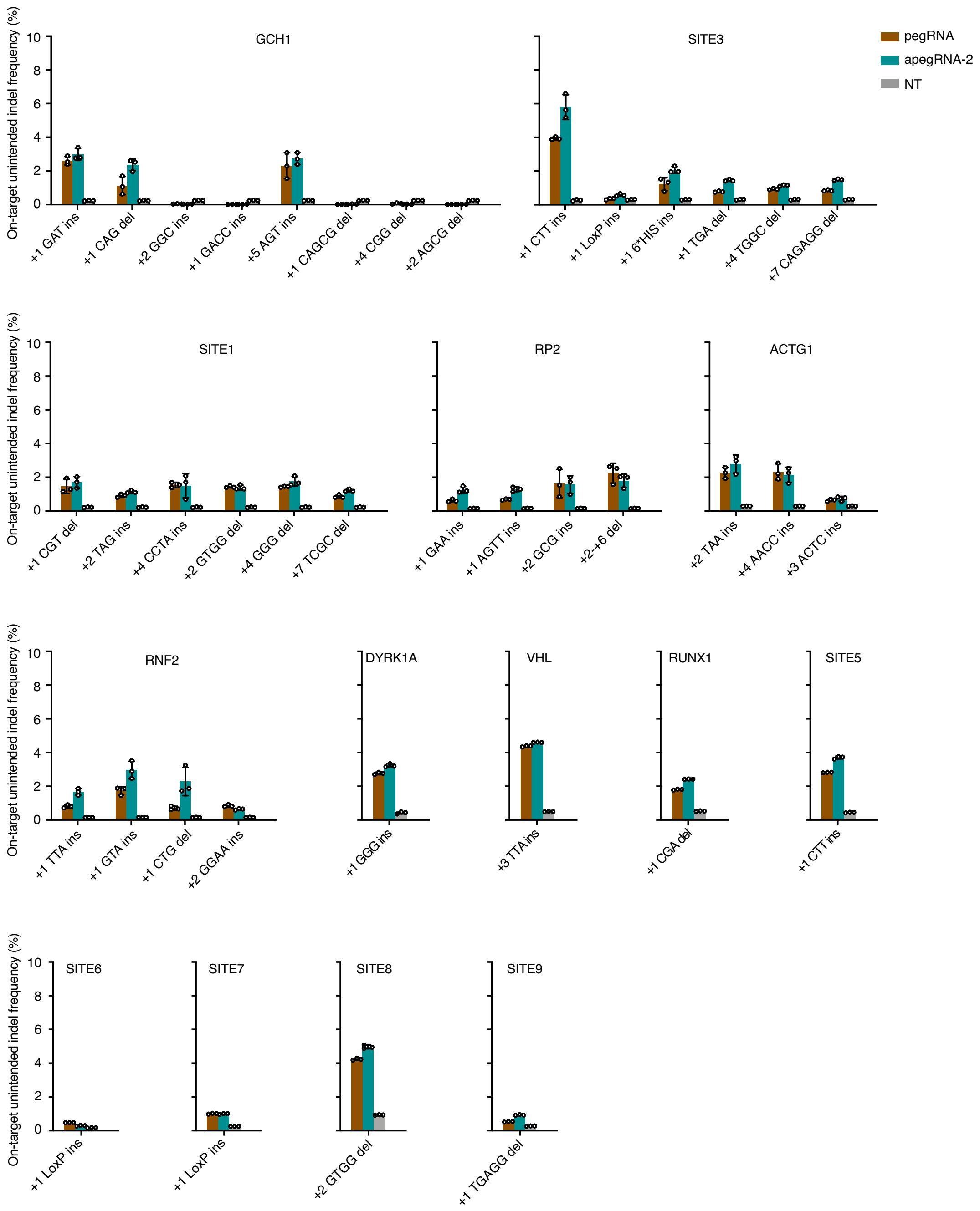

Supplementary Fig. 12. Unintended indels induced by regular pegRNA and apegRNA-2 at on-target sites.

On-target unintended indel frequencies induced by the regular pegRNA and apegRNA-2 at the indicated sites under PE3 setting. Means ± s.d. are from three independent experiments. Source data are provided as a Source Data file.

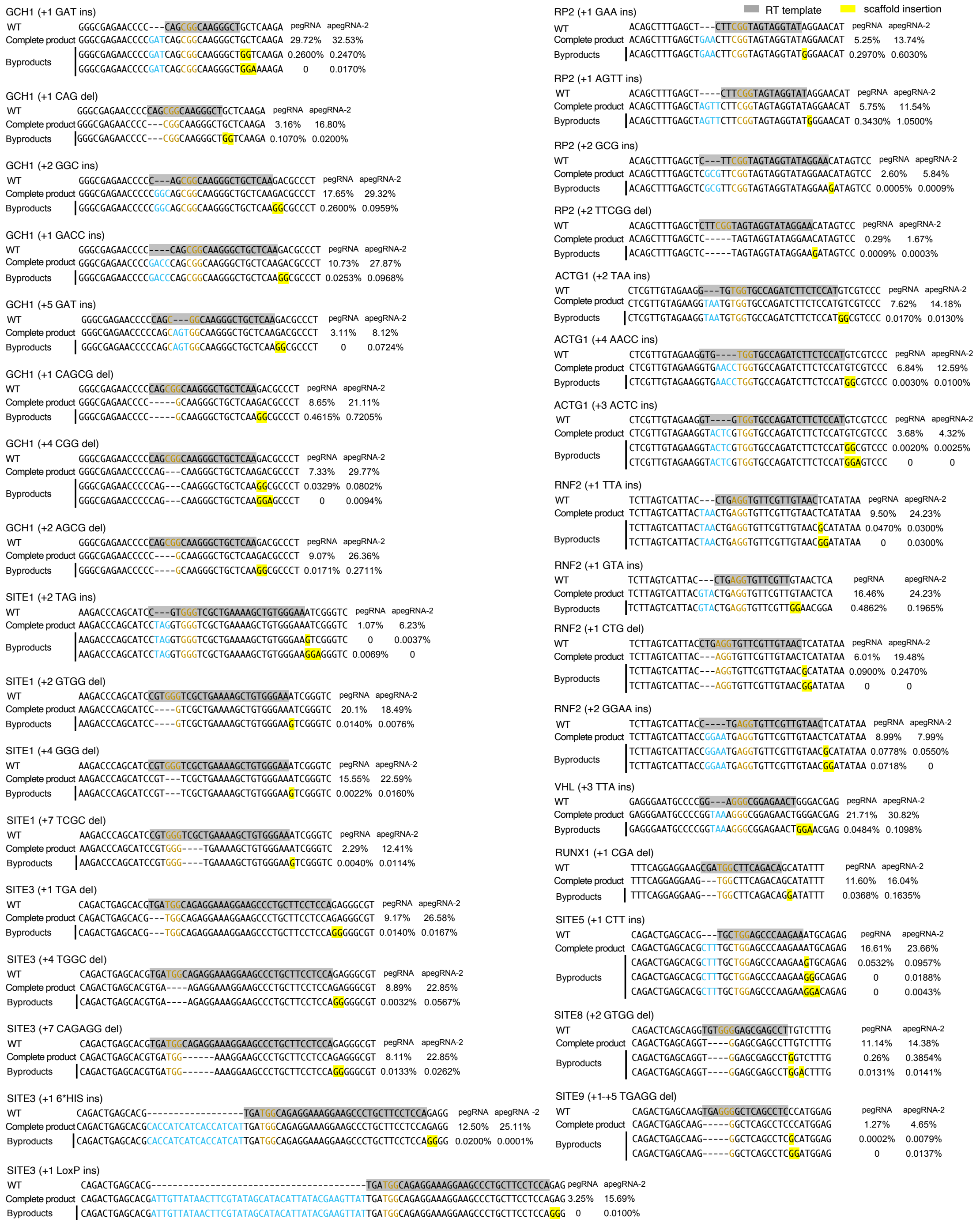

Supplementary Fig. 13. Byproducts induced by regular pegRNA and apegRNA-2 at on-target sites.

The frequencies of complete products and byproducts (with pegRNA scaffold incorporation) induced by the regular pegRNA and apegRNA-2 in Fig. **2b**. The frequencies are means from three biologically independent experiments.

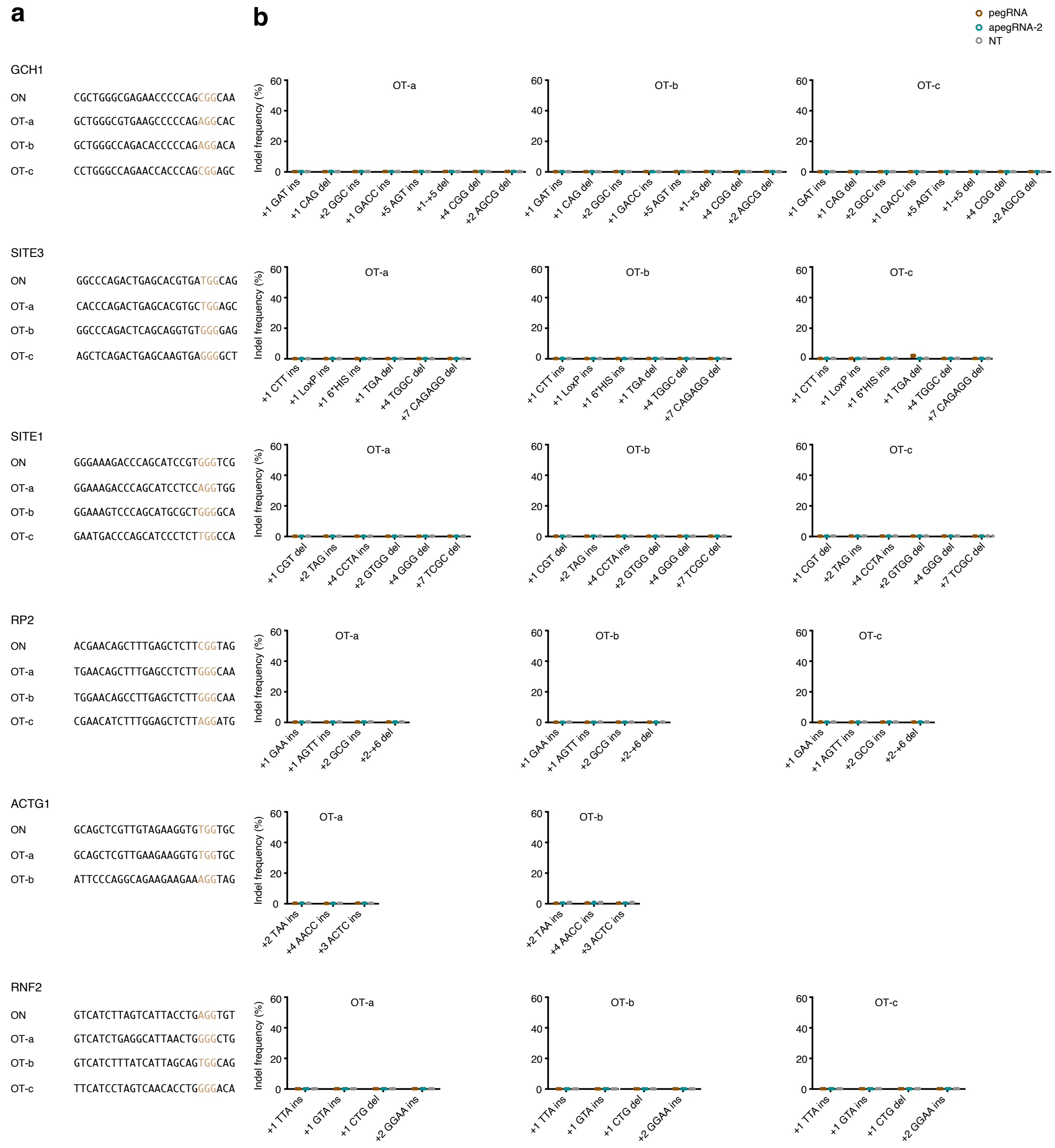

Supplementary Fig. 14. Off-target editing induced by regular pegRNA and apegRNA-2.

(**a**) The sequences of on- and off-target sites for the pegRNAs in (**b**). (**b**) The indel frequencies were determined at the predicted off-target sites. 293FT cells were either treated with the regular pegRNA, apegRNA-2 or left non-transfected (NT). Means ±

s.d. are from three independent experiments. Source data are provided as a Source Data file.

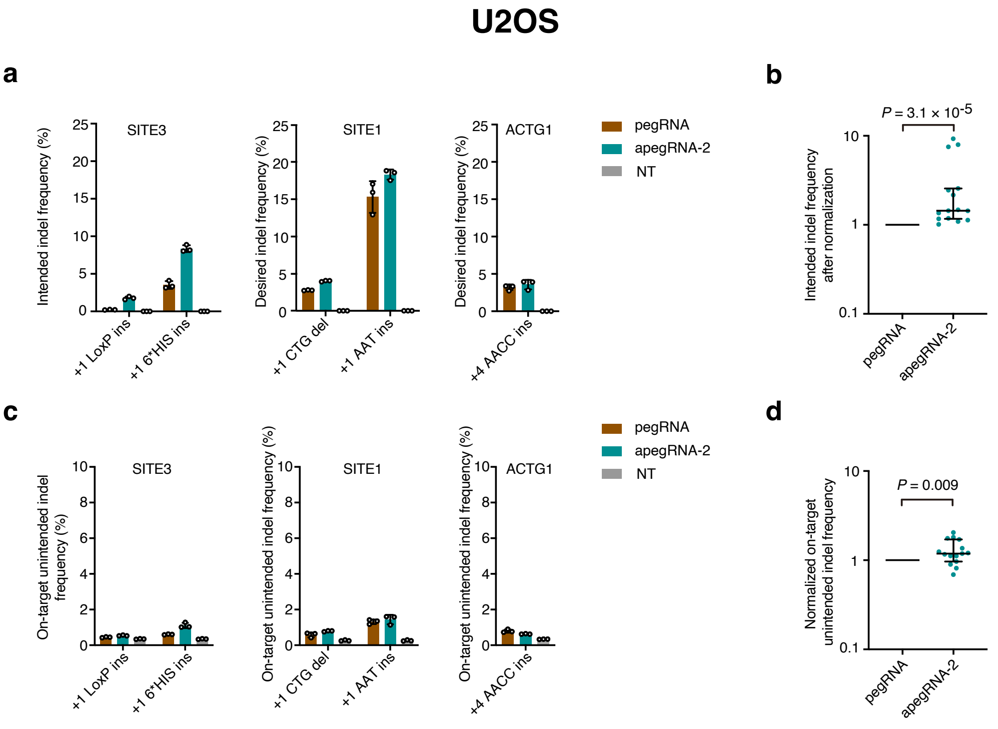

Supplementary Fig. 15. apegRNA-2 induced higher intended indel efficiencies than regular pegRNA in U2OS cells.

(**a**) On-target intended indel editing frequencies induced by the regular pegRNA and apegRNA-2 at the indicated sites in U2OS cells. (**b**) Statistical analysis of normalized editing frequencies, setting the frequencies induced by regular pegRNAs as 1. n = 15 editing from three independent experiments shown in (**a**). (**c**) On-target unintended indel frequencies induced by the regular pegRNA and apegRNA-2 at the indicated sites in U2OS cells. (**d**) Statistical analysis of normalized indel frequencies, setting the frequencies induced by regular pegRNAs as 1. n = 15 editing from three independent experiments shown in (**c**). (**a**, **c**) Means ± s.d. are from three independent

experiments. (**b**, **d**) *P* value, Wilcoxon one-tailed signed-rank test. The median and interquartile range (IQR) are shown. Source data are provided as a Source Data file.

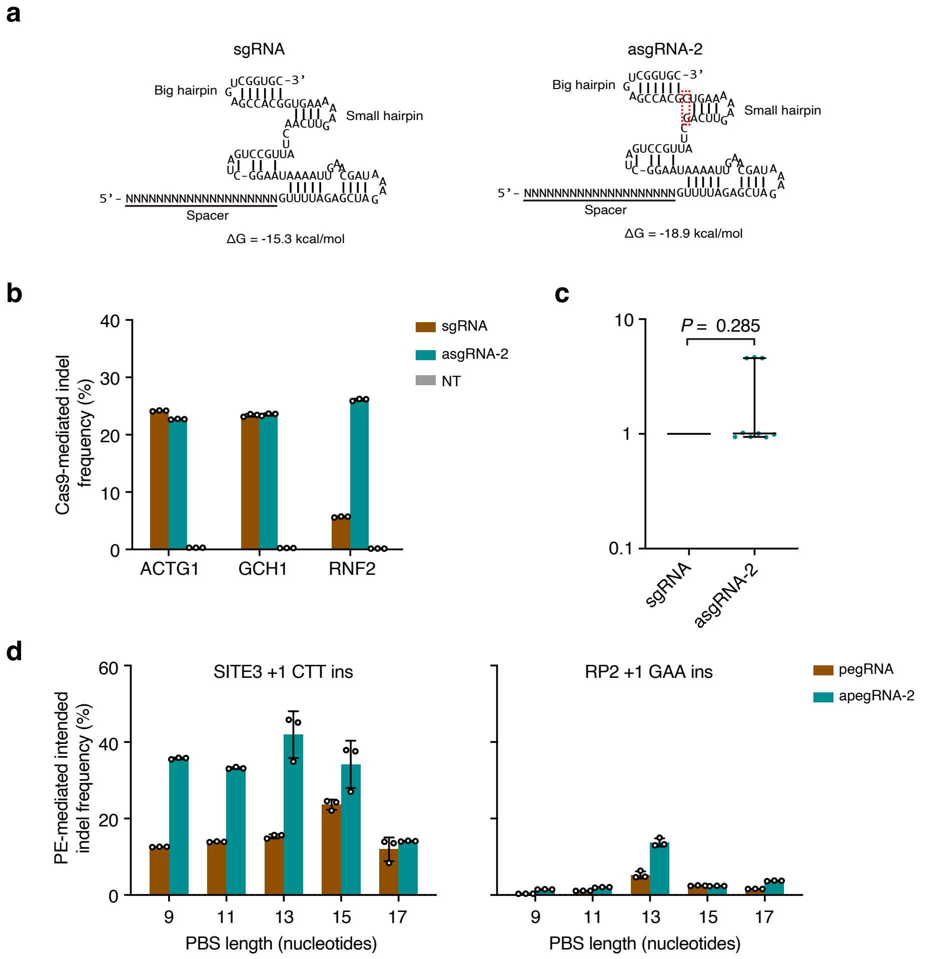

Supplementary Fig. 16. The effect of sgRNA scaffold structure on Cas9-mediated gene editing efficiency and the effect of apegRNA-2 PBS length on PE3-mediated prime editing efficiency.

(**a**) Schematic diagrams illustrating the predicted secondary structures of the regular sgRNA and asgRNA-2, which contains the same small hairpin as apegRNA-2. (**b**) On-target indel frequencies were induced by the co-expression of Cas9 nuclease and the regular sgRNA or asgRNA-2. (**c**) Statistical analysis of normalized indel frequencies, setting the frequencies induced by the regular sgRNA as 1. n = 9 editing from three independent experiments shown in (**b**). *P* value, Wilcoxon one-tailed

signed-rank test. The median and interquartile range (IQR) are shown. (**d**) The intended indel editing frequencies induced by the regular pegRNA and apegRNA-2 with different length of PBS at the indicated target sites. (**b**, **d**) Means ± s.d. are from three independent experiments. Source data are provided as a Source Data file.

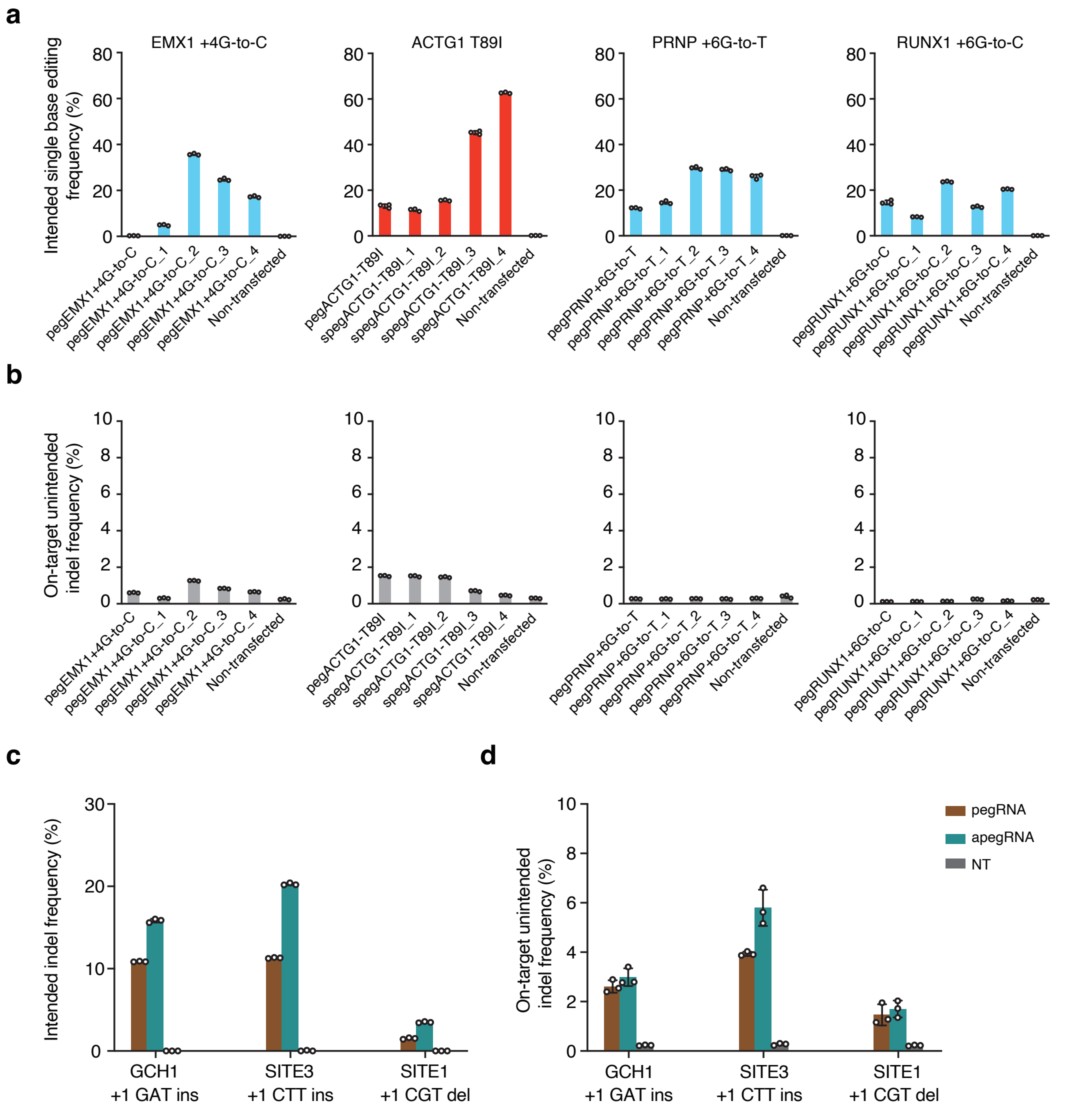

Supplementary Fig. 17. Efficacy of spegRNA and apegRNA in PE2 system.

(**a**, **b**) The intended single-base editing frequencies (**a**) and unintended indel frequencies (**b**) were induced by the regular pegRNA and the pegRNAs containing additional base substitutions under the PE2 setting. (**c**, **d**) Intended indel editing frequencies (**c**) and unintended indel frequencies (**d**) induced by the regular pegRNA and apegRNA at the indicated target sites under the PE2 setting. Means ± s.d. are from three independent experiments. Source data are provided as a Source Data file.

Uncropped gels for Supplementary Fig. 6b

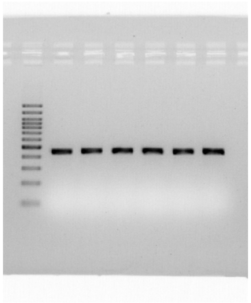 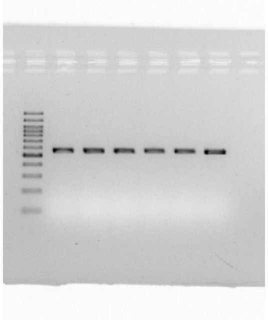

Uncropped gels for Supplementary Fig. 6d

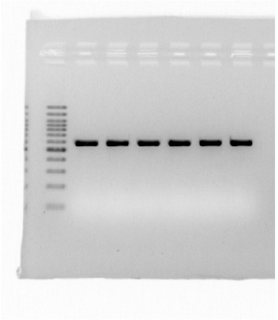 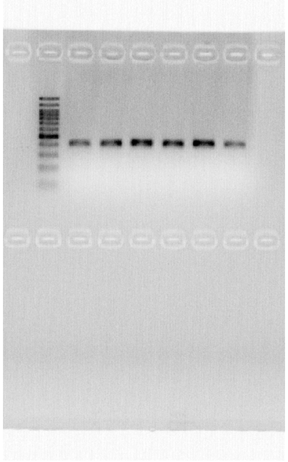 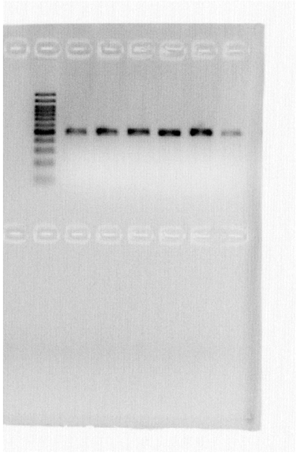

Uncropped gels Supplementary Fig. 6.
