# Supplementary material for: Highly efficient prime editing by introducing same-sense mutations in pegRNA or stabilizing its structure
Source: Nat Commun. 2022 Mar 29;13:1669. doi: 10.1038/s41467-022-29339-9 (PMC8964725; doi:10.1038/s41467-022-29339-9)
Supplement: Supplementary file 2 — Reporting Summary [file 41467_2022_29339_MOESM2_ESM.pdf]

nature research

Corresponding author(s): Jia Chen, Li Yang, Xiaodong Sun, Xiaosa Li

Last updated by author(s): Mar 2, 2022

# Reporting Summary

Nature Research wishes to improve the reproducibility of the work that we publish. This form provides structure for consistency and transparency in reporting. For further information on Nature Research policies, see our Editorial Policies and the Editorial Policy Checklist.

## Statistics

For all statistical analyses, confirm that the following items are present in the figure legend, table legend, main text, or Methods section.

| n/a | Confirmed | |
|---|---|---|
| ☐ | ☒ | The exact sample size (*n*) for each experimental group/condition, given as a discrete number and unit of measurement |
| ☐ | ☒ | A statement on whether measurements were taken from distinct samples or whether the same sample was measured repeatedly |
| ☐ | ☒ | The statistical test(s) used AND whether they are one- or two-sided<br>*Only common tests should be described solely by name; describe more complex techniques in the Methods section.* |
| ☒ | ☐ | A description of all covariates tested |
| ☒ | ☐ | A description of any assumptions or corrections, such as tests of normality and adjustment for multiple comparisons |
| ☐ | ☒ | A full description of the statistical parameters including central tendency (e.g. means) or other basic estimates (e.g. regression coefficient) AND variation (e.g. standard deviation) or associated estimates of uncertainty (e.g. confidence intervals) |
| ☐ | ☒ | For null hypothesis testing, the test statistic (e.g. *F*, *t*, *r*) with confidence intervals, effect sizes, degrees of freedom and *P* value noted<br>*Give P values as exact values whenever suitable.* |
| ☒ | ☐ | For Bayesian analysis, information on the choice of priors and Markov chain Monte Carlo settings |
| ☒ | ☐ | For hierarchical and complex designs, identification of the appropriate level for tests and full reporting of outcomes |
| ☒ | ☐ | Estimates of effect sizes (e.g. Cohen's *d*, Pearson's *r*), indicating how they were calculated |

*Our web collection on statistics for biologists contains articles on many of the points above.*

## Software and code

Policy information about availability of computer code

| | |
|---|---|
| Data collection | Sequencing data were acquired using an Illumina HiSeq X10 (2×150) or NovaSeq 6000 (2×150). |
| Data analysis | DNA deep sequencing read quality was evaluated by FastQC (v0.11.8) and low quality read sequences were trimmed. BWA-MEM algorithm (0.7.17-r1188) was used to map trimmed reads to the targeted sequences. Base substitution calling was performed by SAMtools (v1.9) mpileup function and indel calling was based on the CIGAR values of the SAM/BAM files. All statistical analyses were performed with R (v4.1.1, http://www.R-project.org). The custom Perl and Shell scripts for calculating frequencies of base substitutions and indels (CFBI) are available at GitHub (https://github.com/YangLab/CFBI, v1.0.0). |

For manuscripts utilizing custom algorithms or software that are central to the research but not yet described in published literature, software must be made available to editors and reviewers. We strongly encourage code deposition in a community repository (e.g. GitHub). See the Nature Research guidelines for submitting code & software for further information.

## Data

Policy information about availability of data

All manuscripts must include a data availability statement. This statement should provide the following information, where applicable:

- Accession codes, unique identifiers, or web links for publicly available datasets
- A list of figures that have associated raw data
- A description of any restrictions on data availability

The deep sequencing data generated in this study can be accessed in Gene Expression Omnibus under the accession code GSE197730 (https://www.ncbi.nlm.nih.gov/geo/query/acc.cgi?acc=GSE197730, with the token: ijolkggkjrsdzkj)and in National Omics Data Encyclopedia under the accession code OEP003181 (https://www.biosino.org/node/project/detail/OEP003181). The processed data about all base substitution frequencies and indels frequencies are provided in Supplementary Data 3-5. Source data are provided with this study. All other data supporting the finding of this study are available from the

corresponding author on reasonable request.

# Field-specific reporting

Please select the one below that is the best fit for your research. If you are not sure, read the appropriate sections before making your selection.

☒ Life sciences ☐ Behavioural & social sciences ☐ Ecological, evolutionary & environmental sciences

For a reference copy of the document with all sections, see nature.com/documents/nr-reporting-summary-flat.pdf

# Life sciences study design

All studies must disclose on these points even when the disclosure is negative.

| | |
|---|---|
| Sample size | No statistical methods were used to predetermine sample size. Experiments were independently performed three times unless indicated. In previous studies using related experiments, the sample size has been determined to be sufficient to ensure reproducibility. |
| Data exclusions | No data were excluded. |
| Replication | Experiments were performed three times independently unless indicated. The exact replication numbers were stated in figure legends. The experimental findings in all figures were reproduced successfully. |
| Randomization | Samples were not randomized. The experimental work-flow in this study did not allow/need randomization. |
| Blinding | The investigators were not blinded to group allocation. Blinding is not relevant to our study because it is not a subjective trial and the results presented here are based on objective description of our technology. |

# Reporting for specific materials, systems and methods

We require information from authors about some types of materials, experimental systems and methods used in many studies. Here, indicate whether each material, system or method listed is relevant to your study. If you are not sure if a list item applies to your research, read the appropriate section before selecting a response.

## Materials & experimental systems

| n/a | Involved in the study | |
|---|---|---|
| ☒ | ☐ | Antibodies |
| ☐ | ☒ | Eukaryotic cell lines |
| ☒ | ☐ | Palaeontology and archaeology |
| ☒ | ☐ | Animals and other organisms |
| ☒ | ☐ | Human research participants |
| ☒ | ☐ | Clinical data |
| ☒ | ☐ | Dual use research of concern |

## Methods

| n/a | Involved in the study | |
|---|---|---|
| ☒ | ☐ | ChIP-seq |
| ☒ | ☐ | Flow cytometry |
| ☒ | ☐ | MRI-based neuroimaging |

## Eukaryotic cell lines

Policy information about cell lines

| | |
|---|---|
| Cell line source(s) | HEK293FT cell line was from Thermo Fisher Scientific, and U2OS and HeLa cell lines were from ATCC. |
| Authentication | No cell line was authenticated. |
| Mycoplasma contamination | Cell lines have been tested negative for mycoplasma contamination by PCR methods. |
| Commonly misidentified lines (See ICLAC register) | No commonly misidentified cell line was used. |
