# Supplementary material for: Highly efficient prime editing by introducing same-sense mutations in pegRNA or stabilizing its structure
Source: Nat Commun. 2022 Mar 29;13:1669. doi: 10.1038/s41467-022-29339-9 (PMC8964725; doi:10.1038/s41467-022-29339-9)
Supplement: Supplementary file 3 — Description of Additional Supplementary Files [file 41467_2022_29339_MOESM3_ESM.pdf]

Title: Supplementary Data 1:
Description: Oligos used for plasmid construction.

Title: Supplementary Data 2:
Description: pegRNA target sequences and PCR primer sequences for genomic DNA amplification

Title: Supplementary Data 3:
Description: Calculation of on-target base substitutions. Base substitutions were determined by deep sequencing and base substitution frequencies at the positions in pegRNA-targeted genomic loci were calculated for indicated conditions. Read counts for all four types of bases are listed.

Title: Supplementary Data 4:
Description: Calculation of on-target indels. Indels were determined by deep sequencing and indel frequencies at the examined pegRNA-targeted genomic loci were calculated for indicated conditions. Counts of indel-containing reads and total mapped reads are listed.

Title: Supplementary Data 5:
Description: Calculation of pegRNA-dependent OT base substitutions and indels. Base substitutions and indels were determined by deep sequencing and total base substitution frequencies and indel frequencies at the examined pegRNAdependent OT genomic loci were calculated for indicated conditions. Read counts for all four types of bases and counts of indel-containing reads and total mapped reads are listed.
